# Dynamic Integration of Reward and Stimulus Information in Perceptual Decision-Making

**DOI:** 10.1371/journal.pone.0016749

**Published:** 2011-03-03

**Authors:** Juan Gao, Rebecca Tortell, James L. McClelland

**Affiliations:** 1 Department of Psychology, Stanford University, Stanford, California, United States of America; 2 Center for Mind, Brain and Computation, Stanford University, Stanford, California, United States of America; Indiana University, United States of America

## Abstract

In perceptual decision-making, ideal decision-makers should bias their choices toward alternatives associated with larger rewards, and the extent of the bias should decrease as stimulus sensitivity increases. When responses must be made at different times after stimulus onset, stimulus sensitivity grows with time from zero to a final asymptotic level. Are decision makers able to produce responses that are more biased if they are made soon after stimulus onset, but less biased if they are made after more evidence has been accumulated? If so, how close to optimal can they come in doing this, and how might their performance be achieved mechanistically? We report an experiment in which the payoff for each alternative is indicated before stimulus onset. Processing time is controlled by a “go” cue occurring at different times post stimulus onset, requiring a response within 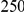 msec. Reward bias does start high when processing time is short and decreases as sensitivity increases, leveling off at a non-zero value. However, the degree of bias is sub-optimal for shorter processing times. We present a mechanistic account of participants' performance within the framework of the leaky competing accumulator model [1], in which accumulators for each alternative accumulate noisy information subject to leakage and mutual inhibition. The leveling off of accuracy is attributed to mutual inhibition between the accumulators, allowing the accumulator that gathers the most evidence early in a trial to suppress the alternative. Three ways reward might affect decision making in this framework are considered. One of the three, in which reward affects the starting point of the evidence accumulation process, is consistent with the qualitative pattern of the observed reward bias effect, while the other two are not. Incorporating this assumption into the leaky competing accumulator model, we are able to provide close quantitative fits to individual participant data.

## Introduction

Imagine you are in a counter-terrorist fight. As a person approaches, you have to quickly identity whether he is a friend or foe and take an action: either you must protect him or kill him before he kills you. The consequences are dramatic and different: the cost is either your own life or your teammate's. How well would you do at making the right move? More specifically, how do we integrate vague stimulus information, such as that person's body-figure, and the consequences of taking each of several possible actions, under time pressure? Can we perform optimally under such circumstances? If so, how is this achieved, and what mechanisms might explain observed deviations from optimality? The answers to these questions tell us more than just how well people can do in such situations. They may also open a window to the underlying mechanism of the interaction between bottom-up stimulus information and higher-level factors such as payoffs.

How observers cope with stimulus uncertainty in decision-making tasks has been intensively studied both experimentally and theoretically [1]–[4]. Models ranging from abstract information processing models to concrete neurophysiological models [1], [2], [5]–[7] agree that the process involves an accumulation of noisy information to drive a decision. However, there has been less emphasis on the question: How do decision makers integrate differential payoffs for responses to the different alternatives? This issue has been explored extensively within the classical literature on signal detection theory [8]–[10], where accuracy and bias without regard to time taken to decide have been the prime considerations. In a dynamic context, there were a few earlier theoretical investigations (See [11], [12] and other papers cited in [12]), but there is only a small and very recent literature combining experimental and computational investigations [12]–[15].

**Figure 1:**
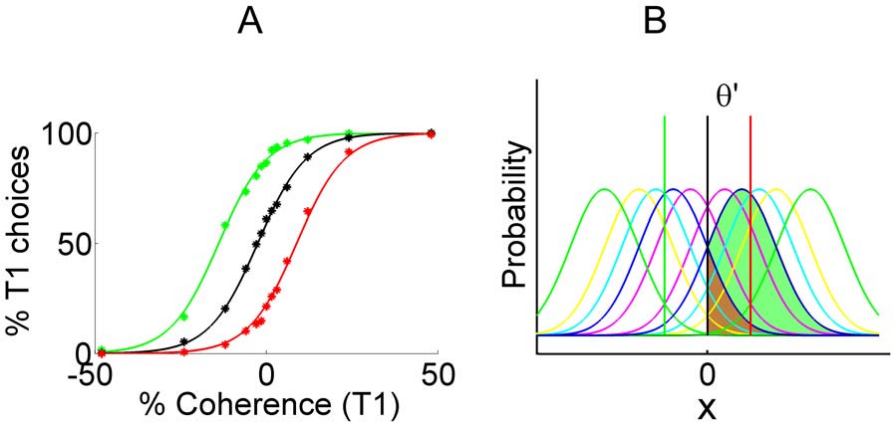
Choice behavior with unbalanced rewards and an account in signal detection theory. A: Response probabilities in a perceptual decision-making task [14] with reward manipulations. Data from one of two monkeys in [14] have been replotted with permission from the authors. Percentage of positive direction choices (denoted 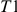 in the figure) increases with motion coherence in the positive direction in a sigmoidal fashion; one direction of motion is nominally defined as positive, the other as negative. Black: balanced reward condition; Green: reward is higher in the positive direction; Red: reward is higher in the negative direction. Dots represent data in [14] and solid curves represent fits based on signal detection theory (SDT) as depicted in panel B. B: a characterization of this choice behavior based on SDT. Gaussian functions in different colors indicate the distribution of the evidence variable 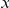 arising in each of the different coherence conditions. Vertical lines indicate the relative positions of the decision criterion. Black, green and red vertical lines represent the criterion positions for the balanced, positive, and negative reward conditions respectively. The area to the right of a specific criterion under a specific distribution corresponds to the percentage of positive choices in that reward and coherence condition. As examples, the areas associated with balanced reward, and coherences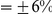 (blue curves) are shaded.

In our work we build on a theoretical analysis [14] of the behavioral data from a recent study in non-human primates [15] investigating the integration of reward and uncertain stimulus information. This study employed a two-alternative forced-choice task with random-dot motion stimuli varying in the percentage of dots moving coherently in either of two directions. Monkeys were trained to judge the motion direction, as in many earlier experiments [3], [16]. In addition, monkeys are informed before motion onset of the amount of reward that would be available for each correct choice (either one or two drops of juice). There was then a 500 msec motion stimulus, followed by a delay of 350–550 msec before the monkeys received a cue to respond. The key behavioral results are shown in Figure 1A. When rewards are balanced, probability of choosing one alternative increases with motion coherence in that direction in a sigmoidal fashion (coherence is treated as a signed quantity with positive numbers representing motion in one direction, called the positive direction, and negative numbers representing motion in the opposite direction, called the negative direction), and is unaffected by the magnitude of the reward. With unbalanced rewards, the sigmoid curve shifts to the left or right, reflecting increased responses to the alternative associated with the higher reward.

In their theoretical analysis of this behavioral data, Feng et.al. [14] found that monkeys are almost optimal in their use of reward information to bias their decisions about uncertain stimulus information. We rely on signal detection theory [17] to capture the pattern of results and to provide a grounding for the analysis of the dynamics of reward processing explored in the present article (our formulation is equivalent to the formulation offered by Feng. et.al. [14] but slightly different in its formalization). In signal detection theory, the presentation of a stimulus is thought to give rise to a normally-distributed evidence variable. The mean value of the evidence variable depends on the stimulus condition; the value on a specific trial is thought to be distributed normally around this mean. Feng et. al. [14] found that a good fit to the data is obtained by treating the mean as linearly increasing with the stimulus coherence, and the standard deviation of the distributions as the same for all values of the coherence variable.

According to signal detection theory, the monkey makes a decision by comparing the value of the evidence variable, here called 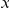, with a decision criterion 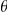. From these assumptions, it follows that the area to the right of 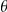 under the distribution associated with each stimulus condition measures the probability of positive choices for that stimulus condition. The effect of reward is to shift the position of this criterion relative to the distributions of evidence values, so that a greater fraction of trials contributing to each distribution fall on the high reward side of the criterion (this could also be achieved by a shift in the evidence variable in the opposite direction). The shift in criterion results in a shift in the sigmoidal curve relating response probability to stimulus coherence, reflecting an increase in the probability of responses in the direction of the more rewarded alternative. See panel B in Figure 1.

**Figure 2:**
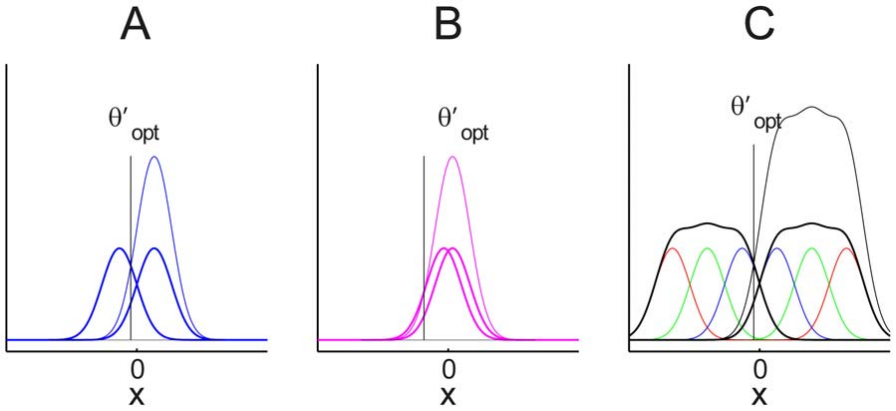
Optimal reward bias for relatively high (panel A), low (B) and combined (C) stimulus levels. A and B: When there is only one stimulus level, the optimal decision criterion is at the point where the distributions intersect after scaling their relative heights by the corresponding reward amounts. The amount of reward bias is smaller when the sensitivity is higher (panel A), and greater when the sensitivity is lower (panel B). C: When multiple stimulus levels are employed, the optimal criterion lies at the intersection of the summed distributions multiplied by the corresponding reward amounts.

Consider a specific pair of coherence values 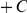 and 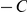, represented by two Gaussian distributions with the same standard deviation. The distance between the two distributions in the unit of their standard deviation is known in signal detection theory as sensitivity, and is called 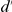. Without loss of generality, we can shift and scale the two distributions so that their midpoint falls at 0 and each has standard deviation equal to 1. In this case their means fall at 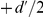 and 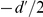 (Figure 2, panel A). The position of the decision criterion, scaled to this normalized axis, represents the degree of bias in units of the standard deviation [10]. Hereafter we will call this the *normalized decision criterion*, and call it 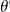. Note that the evidence variable 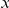 is also a normalized variable.

When payoffs are balanced, signal detection theory tells us that an ideal decision maker should place the criterion at the intersection of the two distributions, i.e. at 0 on the normalized evidence axis. To see why, consider any point to the right of this 0 point. The height of the right-shifted curve indicates the probability of observing this value of 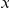 when the motion is in the positive direction 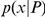, while the height of the left-shifted curve indicates the probability of observing this value of 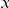 when the motion is in the negative direction 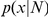. When the two directions of motion are equally likely (as in the experiments we consider here), Bayes' rule immediately tells us that we are more likely to be correct if we choose the positive direction for all points to the right of 0: 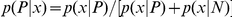 is greater than 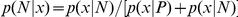. Conversely, we will be more likely to be correct if we choose the negative direction for all points to the left of 0. This shows that the best placement of the decision boundary is right at 0 in this situation; with any other placement our choices would have a lower overall probability of being correct.

When the payoffs are unbalanced, we assume the participant is seeking to maximize the expected reward. The expected value of each choice is equal to the probability that the response is correct, times the reward value of this response. The relative expected value of the two alternatives at each value of 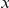 can be illustrated graphically by scaling the distribution functions. We illustrate this in Figure 2A for the case where the reward for a response in the positive direction is twice as large as the reward for a response in the negative direction.

With this scaling included in the heights of the curves, these heights now represent the relative expected value of the positive or negative choice for each value of the normalized evidence variable 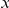. These heights tell us, for example, that if the value of the evidence variable sampled on a particular trial falls right at 0, the expected reward will be maximized by choosing the positive response, because the height of the right-hand curve is higher at this point than the height of the left-hand curve. As before, the best choice of the placement of the criterion is to put it at the place where the curves intersect. To the left of this point, the expected payoff is greater for the negative direction; to the right of this point it is greater for the positive direction. As can be seen, this means that the optimal placement of the criterion is shifted to the left, producing an increase in the proportions of the area under the curve to the right of the criterion under both the positive and the negative distributions.

Now we can visualize how the optimal decision criterion is affected by sensitivity. When stimulus sensitivity is low (Figure 2B), the crossing-point of the two curves is shifted further to the left. Indeed, in the extreme case where sensitivity is zero, the expected value is always greater for the higher reward alternative, and so the optimal policy is to always choose the higher reward alternative. On the other hand, when the stimulus sensitivity is very high, the optimal shift becomes very small. The farther apart the two distributions are, the closer to zero is the point where the distributions cross. In fact it is easy to show that the optimal criterion position is inversely proportional to sensitivity:
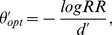
(1)where 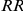 is reward ratio, 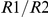.

When multiple stimulus levels are used and randomized in an experiment, the optimal criterion placement depends on exactly the same logic already developed, but is made more complicated by the fact that the stimuli associated with motion in the positive direction now come from several different distributions rather than just a single distribution. The probability of observing a particular evidence value when the direction was positive is the sum of the probability of observing the value under each of the distributions associated with the different positive stimulus levels, normalized to sum to 1; and similarly for the negative direction. For simplicity, the standard deviations of the distributions are all taken to be the same, and the means are assumed to be symmetrically distributed around the 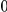 point of the evidence dimension 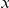. Within these constraints, we can then compute the optimal decision criterion, by locating the position of the intersection of the *summed* distribution functions scaled by the corresponding reward values (Figure 2C), and compare it with the bias observed in each participant's performance to see how close the decision maker comes to being optimal. The Figure gives an example of what the distributions of values of the evidence variable might look like for three positive and three negative stimulus levels whose means are spaced proportionally to the spacing of the physical stimuli used in our experiment.Note that the actual spacing can be determined empirically, and need not be proportional to the physical spacing; remarkably, however, the sensitivity data as shown in [14] are consistent with proportional spacing, and the same holds for the data from the present experiment.

The human and animal psychophysical literature on reward bias [8]–[10] indicates that task details can have a huge impact on measures of bias, and that, in some tasks at least, there are large individual differences between participants. It is all the more remarkable, therefore, that the data reported in Feng et. al. shows a high level of consistency across the two animals, and follows a simple pattern, consistent with a single criterion value for each level of relative reward across all levels of stimulus difficulty. This pattern is consistent with a statement in MacMillan and Creelman [10] that a constant criterion is most likely to be observed when stimuli differing in sensitivity are intermixed, and participants cannot easily discern the relative difficulty level of the stimulus on each trial [18].

Feng et.al. found that for both monkeys, the magnitude of the criterion shift due to the reward manipulation is approximately optimal given the range of stimuli used and their sensitivity to them, deviating very slightly in the over-biased direction for both of the monkeys in the experiment. Once again, this is a simpler and more consistent pattern than the patterns found in other studies [8], [9]. Task variables such as strength of motivation to maximize reward and the provision of accuracy feedback on a trial-by-trial basis may well contribute to the simplicity and clarity of the reward effect in the data reported in Feng et. al.

The results of the analysis in Feng et. al. are encouraging from the point of view of indicating that participants can perform close to optimally under fixed timing conditions, at least under certain task conditions. However, these results leave open questions about whether or to what extent observers can achieve optimality when the time available for stimulus processing varies, so that on different trials participants must respond based on different amounts of accumulated information. This question is important for decision-making in many real-world situations, where the time available for decision-making is not necessarily under the control of the observer, and thus may have to be based on incomplete evidence accumulation. Also, the behavioral results do not strongly constrain possible mechanistic accounts of how observers achieve the near optimal bias they exhibit, as part of a process that unfolds in real time. Indeed, Feng et. al. were able to suggest a number of different possible underlying process variants that could have given rise to the observed results. These issues are the focus of the current investigation.

The empirical question at the heart of our investigation is this: How does a difference in reward magnitude associated with each of two alternatives manifest itself in choice performance when observers are required to make a decision at different times after stimulus onset, including both very short and much longer times? We investigate this matter using a procedure often called the *response signal* procedure, in which participants are required to respond within a very brief time (250 msec) after the presentation of a “go” cue or response signal. Previous studies using this procedure [1], [19], [20] have shown that stimulus sensitivity builds up with time according to a shifted exponential function. That is, when stimulus duration is less than a certain critical time 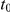, stimulus sensitivity is equal to 0. As stimulus duration lengthens beyond this critical time, sensitivity grows rapidly at first, then levels off. Under these conditions, we ask how effectively participants are able to use differential payoff contingencies. Are participants able to optimize their performance, so that their responses at different times reflect the optimal degree of reward bias? Several delays are used ranging from 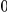 to 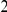 seconds, a time past the point at which participants' performance levels off. Intuitively, (and according to the analysis given above) with zero stimulus sensitivity, at very short delays, an ideal decision maker should always choose the higher reward alternative. As stimulus sensitivity builds up, reward bias should decrease, and level off in an predictable way. Do decision makers achieve optimality when forced to respond at different times after stimulus onset? If not, in what way do they deviate from optimality?

Using the response signal method, we will see that sensitivity grows with stimulus processing time, following a delayed exponential function, consistent with previous studies. We also find that the reward bias, as measured by the position of the criterion 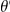, is larger for short stimulus duration and becomes smaller as processing time increases. Although weaker, the reward bias effect is still present even for the longest times, after performance has leveled off. Consistent with [14], we find participants are close to optimal for long processing times, although slightly under-biased unlike the monkeys. For short processing times, however, where stimulus sensitivity is zero, participants are considerably under-biased.

A failure of optimality such as the one we will report invites the question: How can we explain the actual observed pattern of behavior? We explore this question within the context of the leaky competing accumulator (LCA) model [1]. This model is one of a broad class of accumulator models of decision-making (See [4] for a review), incorporating leakage or decay of accumulated information, as well as competition among accumulators, factors motivated both by behavioral and neurophysiological considerations, in the context of a stochastic information integration process. We discuss the behavioral motivation below. Here we briefly note that leakage (or decay) of the state of neural activity and inhibition among populations of neurons are both characteristics of the dynamics of neural processing, and these characteristics were among the key motivating factors behind the development of this model. The model is situated between abstract drift diffusion models [2], [5] and more neurophysiologically realistic models [6], [7]. The presence of inhibition and leakage extend the model beyond the classical drift-diffusion model, though it can be reduced to that model as a special case. Its relative simplicity compared to the more detailed physiological models gives it an advantage in simulation and mathematical analysis. Indeed, the behavioral predictions of the LCA model can be well-approximated under a range of conditions by an even simpler one-dimensional dynamical system called the Ornstein-Uhlenbeck (O-U) process [1], [4], [21], which allows analytical predictions of choice behavior which, we will argue below, increases our insight and facilitates fitting the model to experimental data.

In the LCA model, separate accumulators are proposed for each of the alternative choices available to the decision maker. The accumulators are assigned initial activation values before accumulation begins. At each time step of the accumulation process, a noisy sample of stimulus information is added to each accumulator; the accumulated activation of each accumulator is subject to leakage, or decay back towards an activation of 0, and also to inhibition from all other accumulators. When applied to experiments such as ours, in which a response must be made to a go signal that can come at different times after stimulus onset, the LCA assumes that choice goes to the accumulator with the highest activation value at the moment the decision must be made [1]. When there are two accumulators in the model, choosing the one with the largest activation is equivalent to basing the choice on the difference in activation between the two accumulators: We choose response 1 if the difference is positive, and response 2 otherwise. Because of noise in the evidence accumulation process, this difference variable closely approximates the characteristics of the evidence variable postulated in Signal Detection Theory. Thus, the LCA modeling framework allows us to explore different ways in which reward and stimulus information might be integrated into the decision-making process in real time.

One of the key behavioral motivations for the LCA model was to explain why performance levels off in perceptual decision-making tasks with longer processing times. In the absence of leak or inhibition, the integration of noisy information allows accuracy (measured in 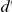) to grow without bound: as accumulation continues, more and more noisy information is accumulated and even a very weak signal will eventually dominate noise. With leakage and/or inhibition, however, sensitivity tends to level off, unless leakage and inhibition are in a perfect balance. When there is an imbalance, performance asymptotes at a level reflecting the degree of imbalance (as well as the strength of the stimulus information), in accordance with the pattern seen in behavioral experiments [1]. Intuitively, with leakage only, older information decays away, preventing perfect integration. Inhibition can counteract the leakage, but if inhibition becomes stronger than leak, early information feeds back through the inhibition and tends to overmatch the influence of later information. We will discuss these points in more detail when we develop the model formally.

While time accuracy curves alone cannot discriminate between leak- and inhibition-dominance, several experiments have now been reported assessing participant's sensitivity to early vs late information. Under conditions like those we use in the present study, in which participants must respond promptly to the occurrence of a go cue, early information tends to be more important than late [22], [23]. Within our framework, this finding is consistent with inhibition dominance, though the authors of [22] prefer an alternative interpretation. With this guidance from other work, we ground our consideration of the mechanism underlying reward effects within the inhibition-dominant regime of the LCA framework, henceforth denoted 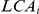.

Using this framework, we test the following hypotheses about the way in which reward information might influence the decision-making process: 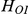: Reward acts as a source of *ongoing input* that affects the accumulators in the same way as the stimulus information, thereby affecting which accumulator has the largest value at the moment of the decision. 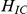: Reward offsets the *initial condition* of the process; it is not maintained as an ongoing input to the accumulators, but it sets the initial state and can therefore influence how the process unfolds. 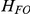: Reward does not enter the dynamics of the information integration process at all, but only introduces a *fixed offset* favoring the accumulator associated with the higher reward. Under both 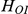 and 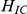, reward input favoring one accumulator will affect the dynamics of the activation process. In contrast, under 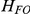, reward does not affect the accumulation dynamics, but only comes into play at the time the choice is made.

Although not exhaustive, these hypotheses encompass three natural ways reward information might enter the decision process. The first two hypotheses were considered in [14], but could not be discriminated; the third one could also have been used to model the monkey behavioral data. The fact that the decision occurred at an approximately fixed time after stimulus onset prevented that experiment from discriminating among these three possibilities. However, the analysis of the neurophysiological data from the same experiment, reported in [15], did provide relevant information: The data provided no support for the idea that reward produced an ongoing input into the accumulators (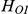), but did provide direct support for the idea that reward affected the initial activation of accumulators at the time stimulus information began to accumulate (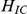). Modeling work reported in that paper indicated that such an offset in the starting activation of the accumulators was sufficient to account both for the physiological data and the behavioral data reported in the paper, without the need to also introduce a shift in the decision criterion (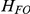).Our theoretical analysis will show that the three hypotheses make distinct predictions about the qualitative changes we should see over time in the magnitude of the effect of reward bias. Thus, as we shall see, our experimental data can be used to provide both a qualitative and a quantitative assessment of the adequacy of each of the three alternative accounts of the possible role of reward in the dynamics of processing within the inhibition dominant leaky competing accumulator model.

Issues similar to the ones we investigate here have also previously been explored in two recent studies [12], [13]. In these studies, participants were required to decide whether two horizontal lines presented to the left and right of fixation were the same or different in length, under different deadline and payoff conditions; as in [15] and in the studies we will report, information about payoffs was presented in advance of the presentation of the stimulus display. In the first of these papers [12], there was a consideration of optimality, and both papers considered a range of possible models that bear similarities to the set of models considered here. These studies provide important information relevant to the questions we address here. In particular, these studies found no support for models in which the reward acts as a source of ongoing input to the accumulators, and favored a model in which processing of reward information preceded, and set the initial state, of an evidence variable prior to the start of processing stimulus information. However, in their framework, which does not include either leakage or inhibition, a shift in starting place is indistinguishable from a change in decision criterion. Thus, their analysis does not distinguish between our 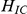 and 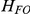 (we will return to a consideration of the models in these papers in the *Discussion* section). Furthermore, the best model they considered, while far better than the others, still left room for improvement in the fit to the data. Thus, it is of considerable interest to explore whether our framework, which includes processes these studies did not consider (specifically, leakage and inhibition), can provide an adequate fit to data from a similar task, and whether the mechanisms offered by our model allow a distinction to be made between 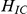 and 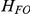. Additionally, it is worth noting two ways in which our study extends the empirical base on which to test model predictions about the time course of reward effects on decision-making. First, our study spans a larger range of processing times, encompassing very short as well as longer times, at which stimulus sensitivity reaches asymptotic levels; and second, each participant in our study completed a substantially larger number of experimental trials, allowing us to assess the adequacy of alternative models to fit individual participant data.

Before proceeding, it is important to acknowledge that there are alternatives to the 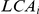 model that could be used to explain some of the important aspects of the data we will report, including the leveling off of accuracy in time-controlled tasks and the relatively greater importance of early- compared with late-arriving information in [2], [22], [24]. Most basically, the leveling off can be explained if there is trial to trial variability in the stimulus information reaching the accumulators [2]. This could either arise because the stimuli themselves vary from trial to trial or because of variation from trial to trial in the output of lower-level stimulus processing processes. In either case, an experimenter's nominal stimulus condition can actually encompass a normally distributed range of effective stimulus values. In this situation, the lossless integration of the classical DDM can eventually achieve a perfect representation of the trial-specific value, but if the distribution of values for different nominal stimulus conditions overlaps, asymptotic sensitivity will remain imperfect. Another way to explain why performance levels off at longer trial durations is to propose that participants do not continue integrating information throughout the entire duration of the trial. Although the response signal method in principle allows participants to continue integrating until the go cue occurs, several authors have proposed that integration may stop when the accumulated evidence reaches a criterial level, even though further integration could result in further improvements in accuracy [22]. With one or both of these extensions of the basic drift-diffusion mechanism it has often been possible to capture the patterns in time controlled data quite well without invoking the leakage or inhibition features of the 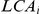. Thus, we offer the analysis we will present here as one possible account for the findings from the present study, though possibly not the only one. We do consider some alternative models in the general discussion and the dataset from our investigation is available for others to use in considering alternative accounts. The data set is available at: http://www.stanford.edu/group/pdplab/projects/GaoEtAlDynamicIntegrationData/.

The rest of this article is organized as follows. Our experiment design is described in *Methods*. The *Results* section contains the results using response probabilities to trace the time-evolution of stimulus sensitivity and reward bias at different times, comparing this with what would be optimal given the corresponding sensitivity. In a third section on *Dynamic Models*, we apply the LCA model to test our three hypotheses about how reward affects the decision-making progress. Finally we return to the broader issues in the *Discussion*.

## Results

### Basic Findings

**Figure 3:**
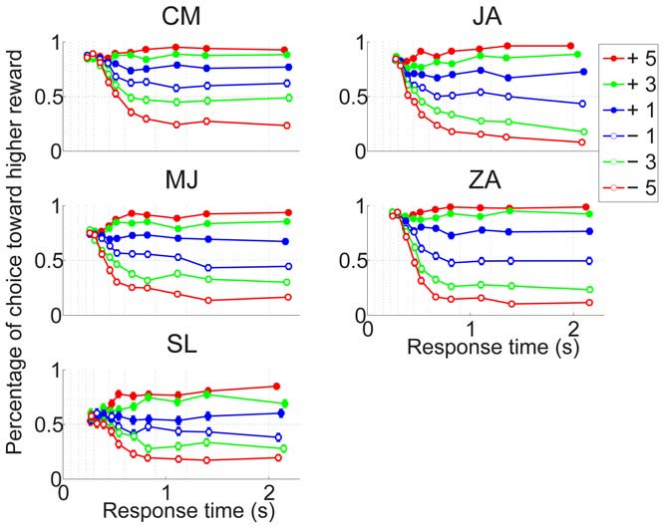
Results of our perceptual decision-making task with unequal payoffs. For each combination of stimulus and delay conditions, the percentage of choices towards higher reward (ordinate) is plotted against the mean response time, the time from the stimulus onset (time 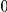) to a response (abscissa). Lines with filled symbols denote congruent conditions in which stimulus and reward favor the same direction, lines with open symbols denote incongruent conditions in which stimulus and reward favor opposite directions. Task difficulty is color coded: Red, green and blue for high, intermediate and low discriminability levels respectively. Dashed vertical lines indicate the time of the “go” cue: 0–2000 msec after the stimulus onset.

To focus analysis on the effect of reward, we collapse across left and right sides and present results in terms of choices toward the *higher* reward alternative. There are hence six stimulus conditions (three amounts of shift towards the higher reward, and three shift amounts toward the lower reward) and ten delay conditions, amounting to sixty combinations. Our observations are summarized in Figure 3. For each combination, we plotted the percentage of choices towards the higher reward *vs* the mean response time for trials in the specified condition. Response time is defined as the time from stimulus onset to a response, equal to the sum of the go-cue delay plus the time to respond from the go-cue delay to the actual occurrence of the response. Lines with filled symbols represent congruent conditions in which stimulus and reward favor the same direction, while lines with open symbols are used for incongruent conditions where stimulus and reward favor opposite directions. For congruent conditions, the probability of choosing the higher reward corresponds to accuracy (proportion correct). For incongruent conditions, proportion correct is 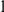 minus the probability of choosing the higher reward.

As in a previous study using a similar method (Experiment 1 in [1]), participants responded promptly to the go cue overall, though all participants' responses were slower when the go cue delay was shorter. This can be seen by measuring the distance along the 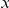 axis from the go cue delay value (successive vertical lines on the figure, starting at 0) to the corresponding data point in the figure. For the shortest go cue delay, participants missed the response deadline 20% to 75% of the time. Rate of missing the deadline declined rapidly at first then leveled off at longer go cue delays. In the longest delay conditions participants missed the deadline 2% to 10% of the time.

All participants' performance, except that of SL, shares the following features: 1) the overall probability of choosing the higher reward, roughly indicated by the mean position of all the curves, is larger for short delay conditions and remains above 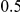 for all delay conditions; 2) The curves for all stimulus conditions all fall on top of each other for the shortest delay condition, indicating zero stimulus sensitivity; 3) Although the responses are completely insensitive to the stimulus at shortest delays, participants do not always choose the higher reward alternative; 4) The curves diverge as processing time increases, tending to level off at long durations. For participant SL, although the curves do diverge as processing time increases, and level off at long durations, there is little or no indication of a bias toward the higher reward, with the possible exception of a very slight deflection in the direction of higher reward for responses in short delay conditions.

### Extracting Sensitivity and Criterion Placement By Delay Condition

The previous section qualitatively answered some of the questions raised in the Introduction: Most participants do exhibit a gradual reduction in the magnitude of the reward bias. To quantify how they deviate from optimality and to motivate dynamic models, we measured their stimulus sensitivity and reward bias separately according to the Signal Detection Theory analysis described in the Introduction. For each delay condition, we calculated three sensitivities 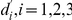 for the three stimulus levels and one value for the normalized decision variable, 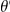, as discussed in the introduction, choosing values that maximize the probability of the data for that delay condition. It should be noted that the adequacy of such an analysis even as a descriptive characterization of the data is not guaranteed, as discussed in the introduction. We assessed this using a graphical method discussed in [10], together with *Chi square* tests. The results of this analysis are presented in Supporting Information S1. The conclusion from this analysis is that, indeed, the three 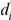 values and single 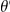 value provide a good empirical description of the data; as in [14], it appears that participants did not adapt their criterion placement as a function of the stimulus difficulty level, as expected when stimulus difficulty varies unpredictably from trial to trial, as it does in our experiment [10], [18].

### Stimulus Sensitivity Analysis

**Figure 4:**
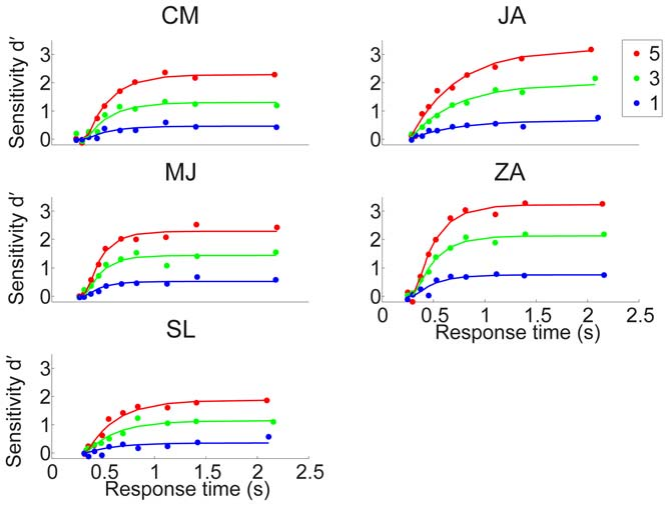
Stimulus sensitivity follows a shifted exponential approach to asymptote as processing time increases. Colors code the three discriminability levels: red, green and blue for 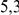 and 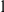 pixel(s) difference respectively. Symbols denote data (see text for details) and solid curves denote the delayed exponential fit.

Sensitivity values as a function of time are shown in Figure 4 (symbols). Apparently stimulus sensitivity grows with stimulus duration initially and then levels off for all participants. To further demonstrate that the sensitivity observed is consistent with the shifted exponential function as in previous studies [1], [19], [20], we then carried out a maximum likelihood fit assuming sensitivity follows a delayed exponential function
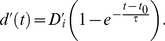
(2)where 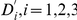 denotes the asymptotic sensitivity levels for the three stimulus conditions, 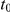 denotes the initial period of time before participants become sensitive to the stimulus and 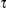 denotes the timescale of the dynamics of the stimulus sensitivity. The fitting results are summarized in Figure 4 (solid curves) and the fitted parameters are summarized in Table 1. The close match between the solid curves and the symbols in Figure 4 suggests that the stimulus dynamics in this experiment is well-captured by the delayed exponential function. We emphasize that sensitivity measures the distance between the centers of the distributions *in the unit of their standard deviation*, and both the mean and the standard deviation of the activation can change over time. Indeed, both variables change in the models we explore in the *Dynamical Models* section.

**Table 1:** Parameters for the delayed exponential fitting.

Participant	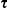	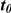	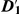	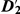	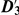
CM	0.23	0.34	0.46	1.3	2.3
JA	0.45	0.27	0.66	2.0	3.2
MJ	0.16	0.34	0.53	1.4	2.3
ZA	0.20	0.32	0.76	2.1	3.2
SL	0.29	0.34	0.35	1.1	1.9

Parameters of the delayed exponential fitting according to signal detection theory. Results for the five participants are shown in five rows. 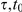 and 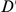 denote the timescale, the delay and the asymptotic value of the delayed exponential function respectively. Subscripts 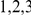 refer to the three stimulus levels. See Equation (2). The fitting result is depicted in Figure 4.

Our experiment employs a simple static visual stimulus, unlike the dynamic motion stimuli used in many primate studies of the dynamics of decision making. Interestingly, however, the time-course of the accumulation of evidence is comparable in our study and the similar study of Kiani et. al. [22], in which standard dynamic motion stimuli are used; in both cases, a time constant on the order of 1/3 of a second appears typical (for one of our participants, the time constant is even longer). This may seem surprising, since in the motion studies evidence must necessarily be integrated over time due to the intrinsic noise of the stimuli, whereas in our study, there is no intrinsic noise in the stimulus. We cannot say, however, whether processing noise arising from micro-saccades or neural sources, or some processing time constant somewhat independent of the noise level, is governing the relatively long time constant seen in our experiment.

**Figure 5:**
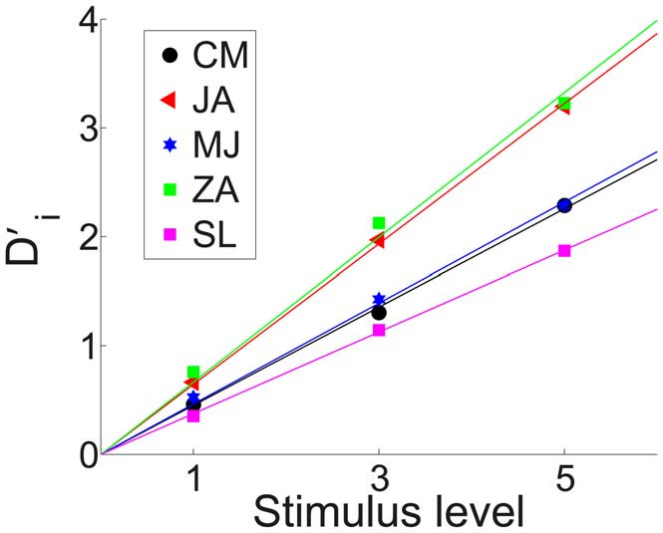
Asymptotic sensitivity scales approximately linearly with stimulus level. Symbols denote the asymptotic sensitivity as in Figure 4 and Table 1; Solid lines denote the linear fit constrained to go through the origin. Fitted values of the scalar 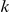 are 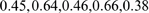 respectively for participants CM, JA, MJ, ZA and SL.

An additional finding that emerges from this analysis is that the asymptotic sensitivity 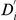 scales approximately linearly with the stimulus level in this study. See Figure 5 for the linear fitting results assuming:
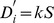
(3)where 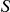 represents stimulus level taking values 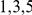 and 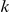 is a linear scalar.

### Reward Bias

**Figure 6:**
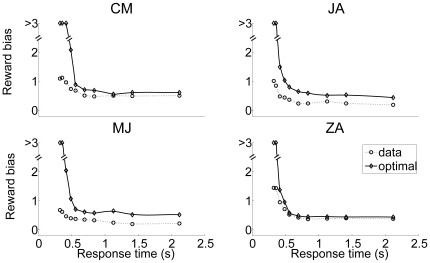
Reward bias is sub-optimal, especially at short delays. The observed reward bias, 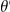 (open circles connected with dotted lines) is put together with the optimal bias 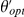 (diamonds with solid curves). Individual panels represent the individual results of the four participants showing a reward bias.

The measured normalized decision criterion, 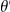, for each delay condition is depicted in Figure 6 (open circles connected with dashed lines). As previously noted, this variable changes in the expected way for all participants except SL, whose behavior is unaffected by the reward manipulation.For each of the remaining participants, we calculated the optimal decision criterion, 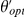, based on the signal detection theoretic analysis presented in the introduction and the observed sensitivity data presented in the preceding section, and plotted these optimal values in Figure 6 (solid curves) together with the normalized criterion value 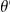 estimated from the data as described above. Note that 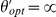 when 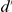 is equal to 0; for display purposes, such values are plotted at an ordinate value of 3.0.

In the calculation of the stimulus sensitivity and the reward bias, 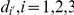 and 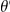, we assumed the distributions of the evidence variables for the three stimulus levels have the same standard deviation: higher sensitivity, associated with higher stimulus levels, results from distributions that are farther apart. However, the increase in sensitivity could result from changes in the standard deviation, as well as the separation of the distributions. Does the finding that participants are underbiased depend on the assumption that the standard deviations are equal? We considered an extreme case in which the sensitivity differences between the different stimulus levels resulted only from a reduced standard deviation, rather than increased separation of the distribution. In this case as well all four participants actual bias came out below what would be optimal; as with the equal standard deviation case, the deviation was larger for short delays and smaller for long delays (results now shown).

To assess the cost of participant's deviations from optimality, we calculated their reward harvest rates: the number of points they obtained relative to the number they could have harvested had they chosen the criterion optimally based on their stimulus sensitivity at each time point. As with the monkeys in [14], all four participants harvested more than 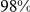 of the points for long delay conditions. For the two longest delay conditions their harvest rates are: 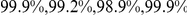. However, for the two shortest delay conditions, the rates are 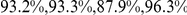 indicating that they are considerably under-biased under these conditions. We consider possible reasons for this underbias in the *Discussion*.

### Dynamical Models

Motivated by the dynamics of the stimulus sensitivity and reward bias, we now explore a possible mechanism underlying the effect of reward on the decision-making process within the context of the leaky competing accumulator (LCA) model. We review the 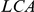 model first and then implement and test the three hypotheses raised in the *Introduction*. This leads to several alternative accounts of the underbiasing of performance on trials at short delays.

### The Leaky Competing Accumulator Model and Its One-Dimensional Reduction

In the leaky competing accumulator model, noisy evidence for each alternative is accumulated over time in each accumulator. The accumulators compete with each other through mutual inhibition, and the accumulated evidence in each is subject to “leakage” or decay. To model our experiment in which participants have to respond promptly after a go cue, we assume that the go cue triggers a comparison of the activation of the two accumulators, and the response associated with the highest value is emitted, subject to a possible offset as discussed below. For our case with two alternatives, the accumulation dynamics is described by
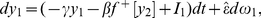
(4)

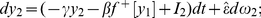
(5)where 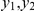 represent the activations of the accumulators, 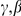 are leak and inhibition strengths respectively, 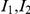 are stimulus inputs to the two accumulators, 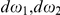 denote independent white noise with strength 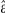, and 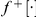 is a nonlinear input-output function arising from the neural inspiration for the model. A neuron does not send outputs to other neurons when its activation goes below a certain level; above this level, its output can be approximated with a linear function of its activation. Motivated by this fact, we follow [1], [25] in using the threshold linear function. The value of the function 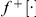 is equal to its argument when the argument is above zero, but is equal to zero when the argument is below zero.

By convention, we treat alternative 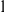 as the positive alternative (associated with the high reward), and alternative 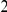 as the negative alternative (associated with the low reward). The assumption that the participant chooses the response associated with the accumulator with the largest activation is equivalent to the assumption that the choice is determined by the sign of the activity difference 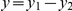 at the moment the accumulators are interrogated. If 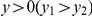, the positive alternative is chosen, otherwise the negative alternative is chosen. Therefore, we only need to track the *difference* between the two accumulators 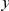, hereafter referred to as the *activation difference variable*. Note that this variable is similar to the normalized evidence variable 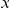 from our analysis using signal detection theory, but is not the same as that variable since it is not scaled in the units of its standard deviation.

As long as the activities of the two units stay above zero, 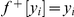, we can subtract Equation(5) from (4), yielding
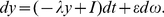
(6)In [1] it was observed that the above simplification can provide a good approximation to the time evolution of the decision outcome of the LCA, as long as the activations of both accumulators are above 0 during the early phases of the information accumulation process (see also [4], [21] and the discussion below for the effect of nonlinearity). In this simplified model, often called the ‘one-dimensional reduction’ of the LCA, the stimulus input 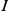 corresponds to the difference between the two stimulus inputs 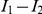. Without reward effects, it should be positive if stimulus 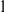 is presented and negative otherwise. In accordance with the approximately linear relationship between the stimulus level and the asymptotic 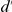 noted in the *Stimulus Sensitivity Analysis* above, we adopt the simplifying assumption that 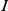 is proportional to stimulus level 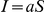 in our primary simulations. The value of the scalar 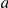, a free parameter of the model, corresponds to the participant's sensitivity to stimulus information. To distinguish this parameter from the sensitivity for a specific stimulus condition, we call it *personal sensitivity*. Noise 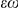 results from the independent Gaussian noise to the two accumulators so that 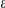 in the one dimensional model is equal to 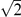 times the value of 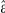 from the two dimensional model. The term 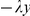 results from the difference in the leak and inhibition in the LCA model 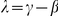.

This one dimension model in Equation (6) is well known as the Ornstein-Uhlenbeck (O-U) process in mathematics and physics, and was first employed in a decision-making context by [26], [27]. Its linear form allows analytical solutions. Before the introduction of reward bias, we follow the natural assumption that the accumulation starts from a neutral state that is subject to trial-to-trial variability. Mathematically, we treat the initial condition 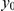 as a Gaussian random variable with zero mean and initial variance 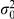. Hence at any time the activation difference variable 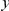 follows a Gaussian distribution with mean and variance
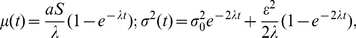
(7)where 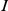 is replaced by 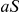 and 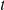 denotes time. When connecting 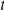 with response times in decision-making tasks, one should acknowledge that it takes time before the stimulus information starts to accumulate, as well as to physically execute the action [1], [2]. We follow the literature and use 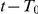 to represent the duration of actual accumulation process, with 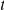 representing total time relative to stimulus onset and 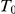 representing the non-decision time just explained.

In general, the value of the activation difference variable 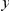 reflects the accumulated noisy signal in the system. The accumulated signal strength is reflected in the mean of 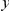 and the accumulated noise strength is reflected in its standard deviation, both of which change over time. In the positive stimulus condition, the mean 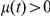, and when the corresponding negative stimulus is presented, the mean 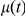 takes the same absolute value but with a negative sign. However, the standard deviation of the accumulated noise, 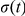, has the same pattern of growth in both cases. The Gaussian distribution with mean 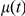 and standard deviation 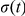 represents the time evolution of the distribution of the activation difference variable across trials for a given stimulus condition. In a particular trial, the activation difference variable is represented as a sample from this distribution, and 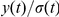 corresponds to the normalized evidence variable 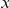 as discussed in the Introduction. Given the assumption that the choice will be positive if 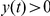, the response probabilities are uniquely determined by the ratio of the mean of the activation difference variable to its standard deviation:
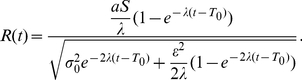
(8)For a specific stimulus condition, for example when the stimulus is shifted three-pixels to the right, this ratio measures the center position of the distribution of the activation difference variable relative to zero in the unit of its standard deviation. Since the mean position of the corresponding opposite stimulus, three-pixel shifts to the left, is the same distance away from zero in the opposite direction, the variable 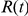 corresponds to half of the stimulus sensitivity 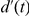 in Signal Detection Theory.

**Figure 7:**
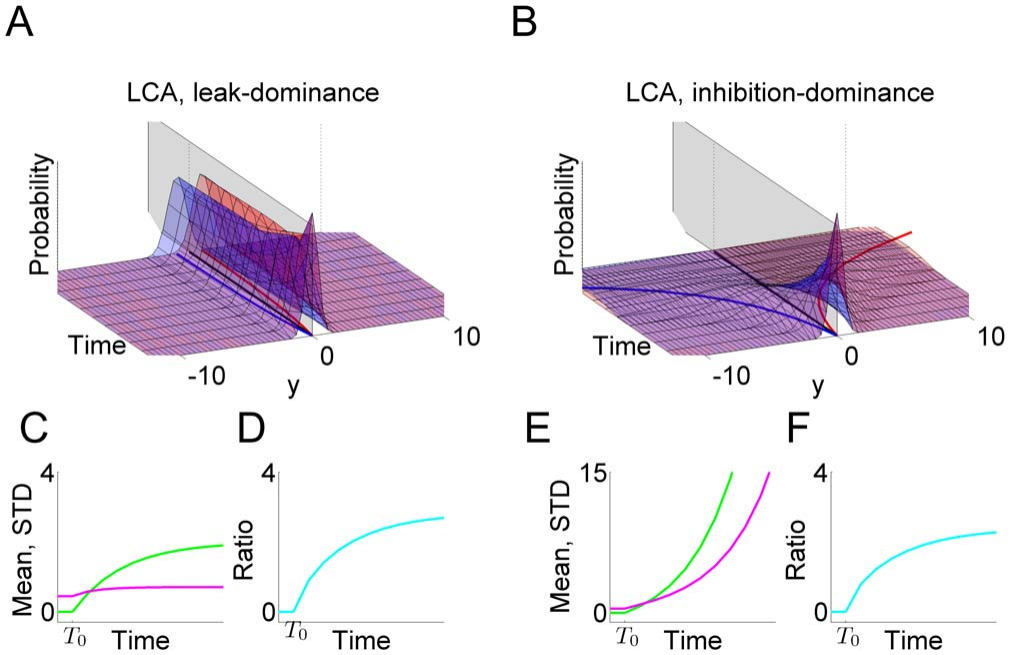
Time evolution of the activation difference variable 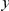 in the reduced leaky competing accumulator model. Top panels: probability density functions of the activation difference variable in leak- (panel A) and inhibition-dominance (panel B). See text for details. At a given time point, the variable is described by a Gaussian distribution (red distribution for a positive stimulus condition and blue for the corresponding negative stimulus). The center position of each distribution (red and blue solid lines on the bottom) represents the mean of the activation difference variable 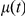 and each distribution's width represents the standard deviation 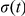. As time goes on, the two distributions broaden and diverge following the dynamics in Equation (7). The distance between them normalized by their width correspond to the stimulus sensitivity 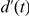, which uniquely determines response probabilities when the decision criterion is zero (vertical black plane). In leak-dominance, the distance between the two distributions and their width (green and magenta lines respectively in panel C) both level off at asymptotic values. In contrast, they both explode in inhibition-dominance (panel E). However, the ratio between the two behaves in the same way (panel D and F). Note: In panels C–F, the 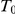 point on the x-axis corresponds to the time at which the stimulus information first begins to affect the accumulators. The flat portion of each curve before that time simply illustrates the starting value at time 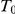.

The stimulus sensitivity predicted by the O-U process above also builds up and levels off with time (see Figure 7D and F), similar to the delayed exponential function used in *Stimulus Sensitivity Analysis*. The closeness of the approximation depends on the value of 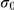, but the shifted exponential provides a good approximation over a range of values of this parameter [1].

The time evolution of the distribution of the activation difference variable 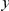 is sketched in Figure 7. We concentrate first on panel A, which represents the time evolution for the case where leak is greater than inhibition, so 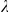 is greater than 0. Here the horizontal axis represents the value of the activation difference variable, and the probability density of it having a particular value is represented in the vertical dimension. Time is depicted moving away from the observer. Red and blue denote two symmetrical stimulus conditions: red for a positive stimulus and blue for the corresponding negative stimulus. The center positions of the distributions (represented by the thick blue and red lines shown on the base plane of each plot) correspond to the mean of the activation difference variable 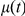 for trials of each type and the width of each distribution represents its across-trial variability 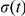. As time goes on, the distributions broaden and diverge symmetrically. Values of the distance between the two center positions (green) and the width of the distributions (magenta) are plotted in panel C, and the ratio between the two, 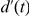 which uniquely determines response probabilities, is plotted in panel D.

As previously noted, panel A of Figure 7 depicts the time evolution of the variable when 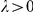. This corresponds to the case where the leakage parameter 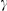 is larger than the inhibition parameter 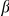 in the underlying two-dimensional LCA model. When 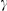 is greater than 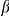, so that 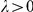, we say the information accumulation process is *leak-dominant*. As [1] noted, in leak-dominance, as noisy information accumulates the effect of any early input decays away. Hence at the decision time, the most recent information plays a larger role.

A very different situation, *inhibition-dominance*, occurs when inhibition is stronger than leak, so that 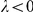. In this situation, whichever alternative has the lead at the beginning tends to dominate and suppress its opponent through inhibition. Earlier information is thus more important in the decision outcome. The mean and the standard deviation of the activation difference variable, although captured by the same equations Equation (7), differ dramatically: they both reach asymptotic values with time in leak-dominance (Figure 7 A), while they both explode to infinity in inhibition-dominance (Figure 7 B). Remarkably, however, the ratio between the two behaves in the same way in the two cases (Figure 7 C and F). Intuitively, the reason for this is that the absolute value of 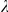 affects the relative accumulation of stimulus information compared to noise in the system. Response probabilities are determined by the ratio between the accumulated signal and accumulated noise, and it is this ratio that behaves the same in the two cases. Indeed, with an appropriate substitution of parameters, exactly the same response probability patterns can be produced in leak- and inhibition-dominance, as discussed in Supporting Information S2. As mentioned in the introduction, however, behavioral evidence from other studies using similar procedures supports the inhibition-dominant version of the LCA model: in these studies, [22], [23] information arriving early in an observation interval exerts a stronger influence on the decision outcome than information coming later, consistent with inhibition-dominance and not leak-dominance. Accordingly, we turn attention to the inhibition-dominant version of the model, and consider the effects of reward bias within this context. We complete the theoretical framework by presenting the predictions in leak-dominance in Supporting Information S3.

Inhibition-dominance is characterized by a negative 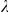 which means the activation difference variable explodes with time (Figure 7B and E). Clearly, this is physiologically unrealistic; neural activity does not grow without bound. However, the explosion is characteristic of the linear approximation to the two dimensional LCA model, and does not occur in the full model itself [1]. In the linear approximation, the explosion is a consequence of the mutual inhibition among the accumulators: As the activation of one of the accumulators goes negative, its influence on the other accumulator becomes excitatory (negative activation times the negative influence results in positive input). However, in the full nonlinear LCA model, when the activity of an accumulator reaches zero, it stops sending any output. The effective inhibition of the other accumulator then ceases, thereby putting that accumulator in a leak-dominant regime, so that its activation tends to stabilize at a positive activation value, while the activation of the other tends to stabilize at a point below 0. (Physiologically, this would correspond to suppression of the potential of the neuron, below the threshold for emitting action potentials.)

**Figure 8:**
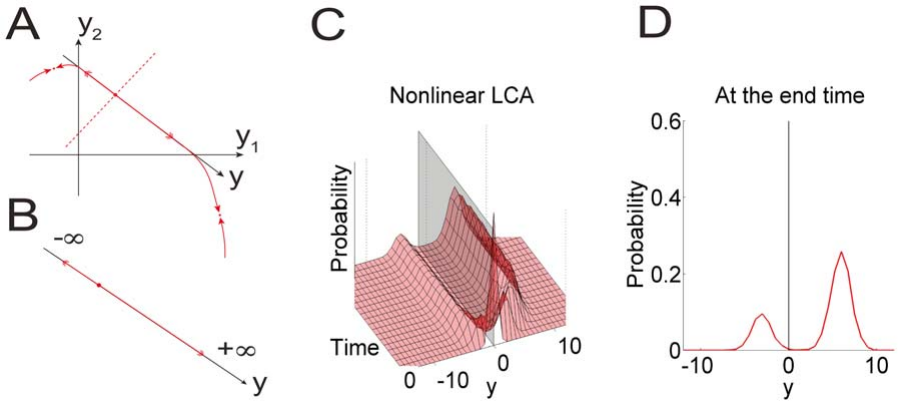
Effect of nonlinearity on the dynamics of the activation difference variable and on response probabilities. Only the case of a positive stimulus is drawn. Left column: phase planes of the full nonlinear leaky competing accumulator model (panel A) and the linear O-U approximation (panel B). In panel A, a point on the 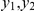 plane represents the two activation variables whose values are read out from the horizonal and vertical axes. The time evolution of the two variables is described by the trace of the point. They explode first until they are out of the first quadrant and then converge to one of the two attracting equilibria. In panel B, the activation difference variable 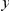 explodes to either 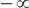 or 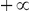. The dashed line in panel A denotes the boundary of the basins of attraction. In the one-dimensional space in panel B, the boundary is denoted by the red dot. Panel C: the probability density function (PDF) of 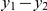 based on the full nonlinear LCA. Panel format is as in Figure 7. Panel D: the PDF at the end of the time interval simulated.

The situation is illustrated in Figure 8A. Here, the dynamics and the two stable equilibria are plotted for a case in which a positive stimulus is presented, favoring accumulator 1. Typically, the accumulators are thought of as being initialized at a point in the upper right quadrant, but as shown in [21] there is a rapid convergence onto the solid red diagonal line illustrated. This diagonal line captures the dynamics of the *difference* between the two accumulators, the activation difference variable 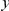 in Figure 8B. Because of the positive input, most trials end in the equilibrium with accumulator 1 active and accumulator 2 inactive (the red point on the bottom right quadrant of the figure), but due to the combined effects of noise in the starting place and in the accumulation process, the network occasionally ends up in a state where accumulator 2 is active and accumulator 1 is inactive (this is the equilibrium point in the upper left quadrant of the figure). The difference between the two accumulators thus diverges at first and then stabilizes near one of two possible values. In the linear Ornstein-Uhlenbeck (O-U) approximation, the difference variable explodes to either positive or negative infinity, as illustrated schematically in Figure 8B. But, since the decision outcome depends on the sign of the difference variable, the linear approximation captures the same decision outcomes as the full nonlinear model, as long as parameters are such that neither activation goes below 0 too early [1], [21].

Panel C of Figure 8 shows the time evolution of the difference between the two accumulators in the full nonlinear LCA model when the positive stimulus is presented as in panel A. The probability of choosing alternative 1 is indicated by the area under the red surface that falls to the right of the black vertical separating plane at 0. With nonlinearity, the distribution exhibits major and minor concentrations corresponding to the two attracting equilibria. This bimodality does not occur without nonlinearity. Instead, the distribution flattens out quickly and its center moves quickly as well (See Figure 7 panel B). However, the areas under the two distributions to the right of the dividing plane can be the same.

Because the one-dimensional O-U approximation allows analytic solutions we use it as a first step in modeling the data. We then present simulations using the full 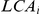 model to confirm that the results are indeed consistent with the underlying model itself, and not only with its one-dimensional approximation.

### Formal Analysis of The Hypotheses for the Effects of Reward

When unbalanced reward is introduced into the 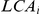 framework, the hypotheses stated in the *Introduction* can be specified as follows. Under the *ongoing input hypothesis*, 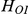, influences of both reward and stimulus accumulate in the same way, so that reward affects the input term 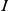, albeit starting before the onset of the stimulus. Under the *initial condition hypothesis*, 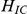, reward information offsets the state of the activation difference variable at the time when stimulus information begins to accumulate, perhaps due to a transitory input ending before the stimulus. Under the *fixed offset hypothesis*, 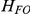, reward information biases decision-making independently of the processes that affect the accumulation of stimulus information. It offsets the activation difference variable by a fixed amount favoring the high reward alternative, or equivalently, it offsets the decision criterion applied to this variable by a fixed amount in the opposite direction. With the help of the Ornstein-Uhlenbeck model, the effect of the reward on the activation difference variable at any time can be quantified under each of these three hypotheses. Without loss of generality, we assume that the higher reward is associated with alternative 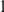, or the positive alternative. So in all hypotheses, the unbalanced reward shifts the activation difference variable in the positive direction relative to the decision criterion in all stimulus conditions.

The *ongoing input hypothesis*, 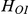, treats reward as an input 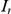 on top of the stimulus input that drives the accumulator. In the full two-accumulator LCA model, this could correspond to an additional input to the higher reward unit resulting in the new input term in the O-U process: 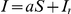. By inserting this to Equation (6), we obtain the new solution for the mean of the activation difference variable
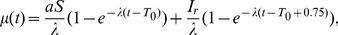
(9)where 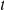 denotes time relative to the stimulus onset. Note that the non-decision time 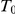 is included in order to match the prediction with response times in the experiment. Comparing this solution with the 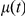 term in Equation (7), one can notice the addition of an independent reward term which grows in the same way as the stimulus. Intuitively, in this hypothesis the activation difference variable is shifted towards the higher reward by an amount that builds up with time. Because the reward cue comes on 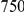 ms before the stimulus, the reward effect is already present to some extent at stimulus onset (note the additional time 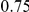 in the reward term), although it will continue to build up further as time continues. The overall strength of reward bias is controlled by free parameter 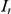.

The *initial condition hypothesis*, 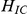, assumes that reward information affects the initial condition or starting point of the process by the amount 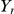. In the full framework of the LCA model, this could result from a higher starting point of accumulator 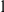, or a lower starting point of accumulator 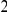, or both. This hypothesis differs from the first one in that reward information enters the accumulation process only at or before the stimulus onset. Mechanistically the reward effect can be thought of as having been subject to integration before the stimulus onset with the integration terminating when the stimulus turns on, or possibly before that time. This effect then follows the dynamics of the system in this hypothesis. Mathematically, the mean of the activation difference variable is changed to
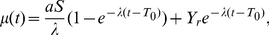
(10)where the dynamic effect of the reward is represented by 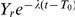. The value of the parameter 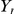 denotes the overall strength of the reward effect.

In the *fixed offset hypothesis*, 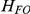, reward affects the decision independently of the sensory accumulation process. The reward effect is therefore treated as a constant offset of the activation difference variable whose mean value is changed to:
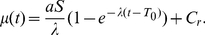
(11)According to this hypothesis, the accumulators only accumulate evidence from the stimulus, and the reward information is essentially processed separately, without interacting with the dynamics of stimulus integration. This is quite different from the situation in the other two hypotheses, where decisions are completely determined by the activity of the accumulator, and reward and stimulus both influence the processing dynamics.

So far, we have quantified the reward effect on the mean of the activation difference variable averaged across trials 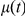. However, response probability is determined by variability 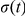 as well as by the mean, as previously discussed. One source of noise is variability in the initial state of the activation difference variable, with standard deviation 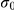. The other source is the noise intrinsic to the dynamics of the process itself, with standard deviation 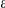. The *absolute* noise level is not measurable in the current experiment because response probability results from the signal to noise ratio. For this reason, we can fix the strength of the intrinsic noise at a specific value, and we set 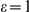 without loss of generality. The fitted values for other free parameters can therefore be viewed as relative to the value of the intrinsic noise level 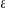.

We now summarize the predictions of the three hypotheses on response probabilities. The probability of choosing the higher reward is determined by the ratio between the mean and the standard deviation of the activation difference variable, which both evolve with time. Thanks to the linearity of the O-U model, it is a linear combination of a stimulus term and a reward term. These hypotheses share the same stimulus term, Equation (8), and they have their unique reward terms
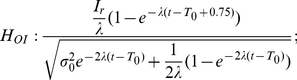
(12)

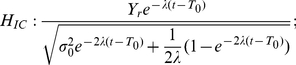
(13)

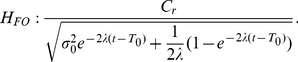
(14)


To see how these hypotheses predict response probabilities as shown in Figure 3, one simply needs to assign values of *S* and *t* to the prediction of a hypothesis, where *S* refers to the stimulus level and *t* refers to the time of a response since stimulus onset. For example, to see the prediction of the ongoing input hypothesis, in the condition of 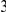 pixels shifted towards the higher reward and responses occurring 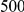 ms after stimulus onset, one should assign 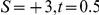 to Equation (8) and Equation (12) to obtain the response probability. The predicted values should be compared with a data point in the corresponding condition in Figure 3 to evaluate the hypothesis. To fit the model to the individual participant data, there are five free parameters that must be fit for each participant. Four of them are shared across the three hypotheses: 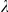 which determines the dynamics of the system (the sign of 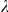 determines whether the process is leak or inhibition-dominant, and its absolute value determines how long the process takes to stabilize); 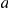, which characterizes the participant's personal stimulus sensitivity; 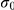 denoting the variability in the initial condition; 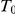, the non-decision time in the task which includes the time it takes before the information arrives at the accumulators and the time for action execution. The fifth parameter is the hypothesis dependent parameter expressing the effect of reward information. In 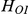, it represents the reward input strength 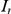; in 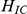 it represents the magnitude of the reward-based offset to the initial condition, 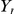; and in 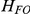, it represents the magnitude of the fixed offset 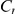.

### Test Results on the Hypotheses

**Figure 9:**
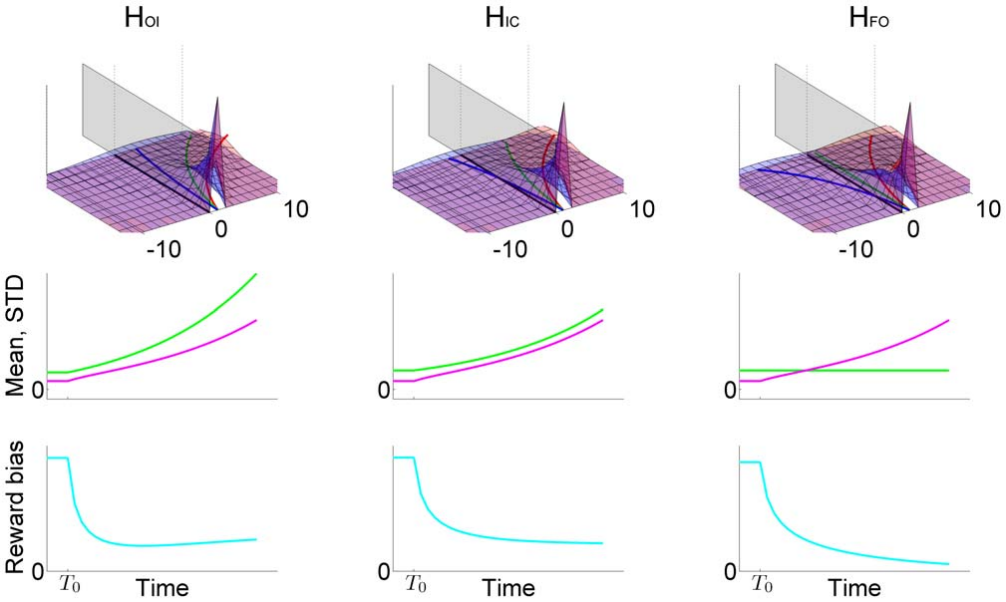
Reward effects in the three hypotheses based on the reduced leaky competing accumulator model. Figure format is similar to that in Figure 7. Top panels: time evolution of distributions of the activation difference variable 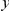 in inhibition-dominance for the positive (red) and negative (blue) stimulus conditions. Solid red, blue and green lines present the mean positions of the positive, negative distributions and the two distributions combined respectively. Middle panels: time evolution of the mean position of the distributions (green lines, as in the top panel) and the standard deviations of these distributions (magenta). Bottom panels: the ratio between the two, which represents the reward bias on the normalized decision variable corresponding to the reward bias in Figure 6. Left column: 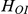, in which reward information provides an ongoing input to the accumulator. Conventions for the x axis in the middle and bottom panels are as in Figure 7. Note that reward cue comes on before the stimulus in the experiment so some reward effect is already present at the stimulus onset. Middle column: 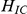, in which reward offsets the initial condition. Right column: 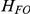, in which reward offsets the activation variable 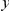 by a fixed amount. Note that under 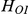, the effect of reward bias on choice grows with time in long delay conditions (bottom left); under 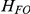 it disappears as stimulus duration lengthens (bottom right); under 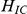, the effect of reward decreases to a fixed value greater than 0 as accuracy reaches asymptote.

The predictions of the three hypotheses are depicted in Figure 9, with each column representing those of each hypothesis. As emphasized before, the analysis focuses on the inhibition-dominant regime in which 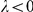. The time evolution of the activation difference variable 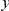 is summarized in the top row. As in Figure 7B, red and blue denote the condition of the positive and negative stimulus respectively. The width of the distributions convey the variability of the activation difference variable, and their center positions, marked by solid red and blue lines below the distributions, indicate their mean values.

Without reward, the distributions are symmetrical (Figure 7B). With a reward influence in place, an overall asymmetry is introduced, corresponding to the reward effect – the time-evolution of the mean reward effect is indicated by the green curve in each panel of the top row of Figure 9. The effect of reward bias on response probability at a given time 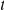 depends on the reward effect on the normalized decision variable, corresponding to the mean of the activation difference divided by its standard deviation. The panels in the middle row show the mean reward effect and the standard deviation of the activation difference variable in green and magenta respectively. The ratio between the two, which represents the qualitative pattern of the normalized reward-bias on response probabilities under each of the three hypotheses, is sketched in the bottom row of the figure and summarized in Equations (12, 13, and 14).

With these figures in front of us, let us now consider the three hypotheses. They all make predictions that are in some ways similar, in that the effect of reward bias starts at a fairly high but finite value, and then drops gradually with time. Focusing first on the starting place and initial drop, these effects arise as follows. Just at the instant that the stimulus effect is about to begin to influence the accumulators (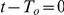), all three hypotheses express the state of the reward bias as a simple ratio of the size of the reward bias that is in effect at that time, divided by the initial variability. In the idealized situation in which there were no such initial variability, then, participants could show the idealized and optimal initial bias, that is, they would always choose the alternative associated with the larger reward. If some initial variability is inevitable, then it is the ratio of the initial bias to the magnitude of this variability that determines how large the reward bias will be. The subsequent drop in the magnitude of the reward bias then reflects, in part, the increase in the overall variance – this increase is the same under all three hypotheses, as illustrated in the middle panels of the figure. As previously discussed, any variability in the activation difference variable at the outset of processing grows exponentially, without limit. This causes the widening and flattening of the distributions in the top row of Figure 9. What differs across the three hypotheses is the way in which the reward bias (captured by the numerators of Equations 12–13) changes as time goes onward.


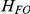
 *: reward as a fixed offset in the value of the activation difference variable*. Under this hypothesis, the reward introduces a fixed offset in the activation difference variable, so that the effect of the reward on the mean of the activation difference variable remains constant over time (green solid line in the middle right panel). Given the increasing variance, the reward effect on choices thus weakens with time when scaled against the accumulated noise. Therefore, the reward effect on response probabilities disappears as stimulus duration lengthens (see bottom right panel in Figure 9). Reviewing Figure 6, we see that the reward bias sustains for long response times. Thus, 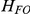 is inconsistent with the data from the participants.


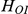
 *vs* 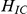 *: reward information participates in processing dynamics*. Under both of the remaining hypotheses, the reward effect on the mean of the activation difference variable grows without limit, but it does so more aggressively in the case where the reward is assumed to provide an ongoing source of input to the accumulators (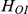, green curve in the middle left panel) than in the case where the reward input only affects the initial conditions of the accumulators (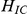, green curve in the middle center panel). At first, under both hypotheses, the dynamics of the normalized decision criterion (i.e. the *reward bias* in the bottom left and center panels) is more affected by the growth of the denominator, causing the ratio to decline. As time elapses, however, the growth of the reward effect under 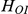 exceeds that of the accumulated noise. The resulting ratio hence starts to grow again. Quantitatively, we can take the derivative of the reward bias with respect to time which indicates that the turn-over occurs at time 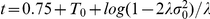. From this we can see that stronger initial variability is associated with an earlier minimum in the value of the normalized reward bias. A similar growing-declining pattern on accuracy was noticed in [28] with dynamical signal strength in the drift diffusion model. The data in Figure 6 indicates that none of the participants exhibited this pattern. Therefore, we conclude that 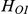 is qualitatively inconsistent with the observed experimental data.

The pattern that we observe under the initial condition hypothesis 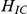 is consistent with the data. In this case, the reward effect on the activation difference variable grows exponentially with time, but it grows more slowly than in 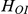, because there is no continuing driving input behind it. The resulting reward bias on choice decreases monotonically with time and levels off, as shown in the bottom middle panel of the figure. Quantitatively, this asymptotic value is equal to 
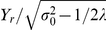
.

### Quantitative Fit Based on 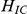


Based on the qualitative superiority of 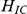, we proceeded to investigate whether a good quantitative fit to the individual participant data could be obtained under this hypothesis. To do so, we assign values of the *stimulus* and *time* to obtain the predicted response probabilities described by Equations(8) and (13). Please see the example below Equation(14). The *stimulus* takes value of 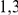 or 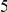 according to the experiment. The value of *time* is the mean reaction time of the participant in a specific experiment condition, defined by the averaged time of the response relative to the stimulus onset. The parameters that were allowed to vary in fitting the data from individual participants were the net inhibition parameter 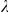 (forced to be negative, in line with the inhibition-dominant regime); the personal stimulus sensitivity 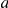; the initial bias strength 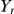, initial variability 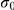, and non-decision time 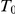. We found values for these parameters that jointly maximize the likelihood of the data, using the MATLAB optimization tool *fminsearch* which finds local minima using the Nelder-Mead simplex algorithm. 50 searches were run for each participant to identify multiple minima and the result with the highest data likelihood was selected.

**Figure 10:**
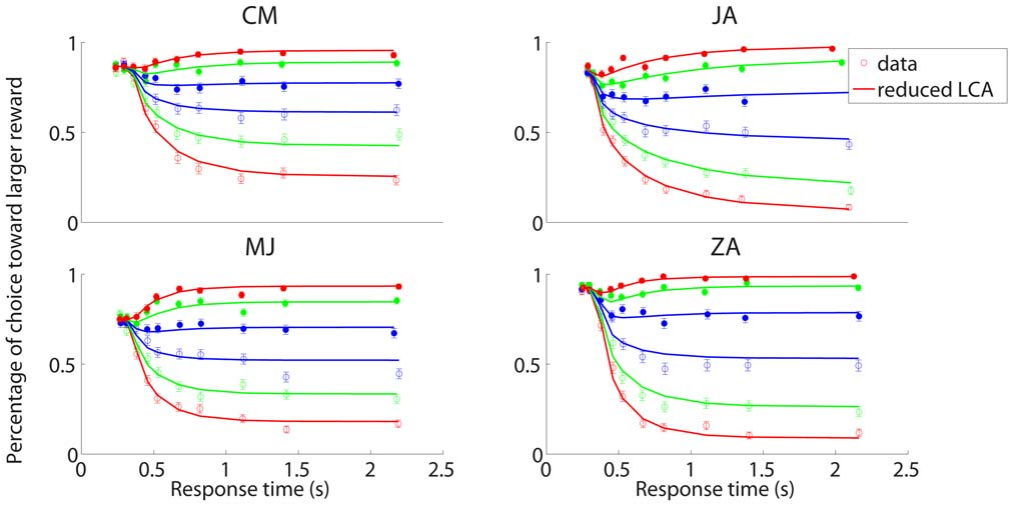
Fitting results under the hypothesis that reward affects the initial conditions of the evidence accumulation process (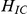), based on the reduced leaky competing accumulator model. Solid curves denote fitting results and symbols denote data as shown in Figure 3. Red, green and blue denote high, intermediate and low discriminability levels respectively. See Table 2 for fitted parameters and log likelihoods. Fitting is based on the one dimensional linear approximation (Ornstein-Uhlenbeck) of the leaky competing accumulator model, see Equation (6).

As before, the intrinsic incoming noise strength 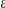 was held constant at 1.0. Parameters 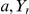 and 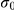, which reflect the activation of the accumulators or their growing rate, are therefore normalized by the noise strength and do not have units. Values of 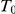 are in *seconds* and of 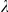 in 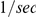. The maximum likelihood values of the parameters are shown in Table 2, and the expected behavioral choice results are displayed in Figure 10. This hypothesis captures all four of the important qualitative features of the data itemized in the section on *Basic Findings*.

**Table 2:** Parameter values in fitting the *reduced* LCA.

Participant	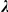	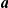	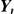	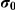	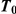	log(p)
CM	−3.4	0.35	0.23	0.21	0.35	−192
JA	−1.6	0.33	0.14	0.14	0.32	−198
MJ	−5.1	0.43	0.10	0.16	0.35	−223
ZA	−3.9	0.52	0.16	0.11	0.36	−199

Fitted parameters of the linear approximation (Ornstein-Uhlenbeck) of the leaky competing accumulator model in the inhibition-dominant regime under the initial condition hypothesis 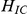. Each row represents the results for each of the four participants who show a reward bias. The model is explained in the main text and summarized in Equations (6, 8, 10, 13). The absolute value of 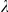 is inversely related to the time scale of the decision-making process and the minus sign means the process is in inhibition-dominance. 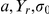 and 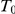 denote a participant's personal sensitivity to the stimulus, the overall strength of the reward bias, the level of the initial variability and the non-decision time respectively.

The correspondence between the experimental data and the model is generally quite close for all four participants. However, there are slight deviations from the fitted values for all four participants. We asked whether the deviation between the data and the model is greater than we would expect by chance by generating 1000 simulated data sets from the predicted response probabilities given by the model, calculating the log likelihood of each such simulated data set, and comparing the value of the log likelihood for the participant's actual data to the distribution of values obtained with the simulated data sets. These simulated values for each participant form unimodal and approximately normal distributions. For two of the participants (CM and JA), the obtained log likelihood falls well within the distribution of values generated by the stochastic simulation (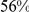 and 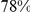 of the simulated values fall below the values for CM and JA respectively). What this means is that, for these two participants, the data are as consistent with the model as we would expect if the model actually generated the data. For the other two participants (MJ and ZA), however, the obtained log likelihood values fall in the tail (below all but 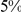 and 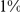 of the simulated values, respectively), suggesting that there may be a real, though subtle, discrepancy between the model and the experimental data. Examination of the relationship between the expected and predicted values in Figure 10 suggests that in the case of participant ZA, the model may be systematically overstating the degree of reward bias in the hardest stimulus conditions (for longer delays, the actual data points for both +1 and −1 conditions tend to fall below the fitted curves for this participant). The pattern of deviations in the case of participant MJ are more scattered, and do not appear to be systematic. We explored the possibility that a better fit to the data for MJ and ZA could be obtained by relaxing the simplifying assumption that the asymptotic sensitivity levels 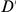 is a linear function of the stimulus level 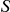. This idea seemed worth exploring because, as can be seen in Table 1 and Figure 5, the approximation seems less adequate for these participants than for the others. However, using the three fitted values of 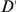 directly, instead of the linear approximation to the relation between 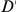 and 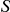, only resulted in a slight improvement in both cases (actual log likelihood values still fall below all but 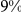 and 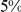 of simulated values based on the direct 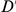 fits for MJ and ZA respectively), and makes the pattern of deviation described in the text for ZA appear even more clearly.

Even if there is room for further improvement in the case of participant ZA, the overall fit of the model to the pattern of the data from all four participants indicates that the model can capture nearly all of the systematic structure in the data.

### Reward Offset in the Full Nonlinear Leaky Competing Accumulator Model

Before we finalize our assessment of the account we have offered for our data, we must revisit the effect of nonlinearity in the full leaky competing accumulator model. To address this we conducted a further fitting exercise using the full LCA. We constrained the parameter values for this fit based on those in the reduced Ornstein-Uhlenbeck model in Table 2. As explained above, the single variable in O-U results from the difference between the two activation variables in the LCA: 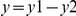, 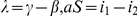 and 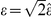. With this relationship, we only have two more free parameters: 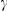 and a baseline input 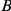. With them, we can specify the strength of the mutual inhibition 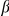 since 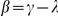, as well as the inputs to the two accumulators: 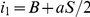 and 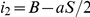. For this simulation, we initialize each of the two accumulators with independent Gaussian random values drawn from distributions with the same mean value 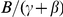 and standard deviation 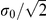. Then, we add half of the initial reward offset amount (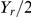) to the first accumulator and subtract this quantity from the second accumulator. Effectively the difference between the two activation variables at the moment stimulus information starts to affect them has a mean of 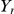 and a standard deviation of 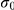. This initial condition corresponds to a two-dimensional Gaussian distribution whose mean falls on the negative diagonal line shown in Figure 8A, shifted along this line toward the direction of higher reward. We then explored possible values of the two remaining free parameters to find values that would allow a good fit to the data.

The chosen values of the parameters are shown in Table 3. Although the parameter 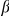 is not independent of other parameters, we show its value in the table as well. Since analytical prediction of response probability is not possible due to the nonlinearity, the values are chosen according to stochastic simulation. For each delay and stimulus condition, we ran 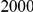 simulated trials. The simulated response probabilities in all the conditions were then treated as the prediction of the model associated with a parameter set. We then searched for a parameter set to maximize the likelihood of the data. These simulations are themselves subject to noise, and there is no guarantee that the best values we found are the best possible values. In fact, similarly good fits can be obtained with other values, as we should expect given previous results demonstrating the adequacy of the one dimensional projection of the model to mimic predictions of the full two dimensional modelUsher2001,Bogacz2006. For given values of the other parameters, the parameter 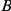 influences the correspondence between the reduced model and the full two dimensional model. When 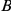 is very large, the correspondence becomes perfect, because both accumulators' activation values stay above 0. As 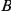 decreases to the point where 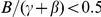, we begin to find subtle effects, whereby occasionally, trials that initially reached the wrong attractor can bounce out of it due to noise, improving accuracy. A subtle effect of this kind may be affecting the simulation results for participants MJ and ZA, but the effect is too small to produce a noticeable improvement in the overall goodness of fit.

**Table 3:** Parameter values in fitting the *full nonlinear* LCA.

Participant	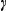	B
CM	1.8	5.3
JA	0.4	1.5
MJ	0.7	2.1
ZA	0.5	2.0

Leak 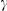 and the baseline input 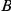 in the full nonlinear leaky competing accumulator model. Based on these parameters and the parameters from the reduced model (Table 2), we can obtain the inhibition strength 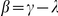 and the inputs to the two accumulators 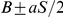 where 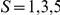 refers to the stimulus levels. See Equations (4–5) and the main text for details.

**Figure 11:**
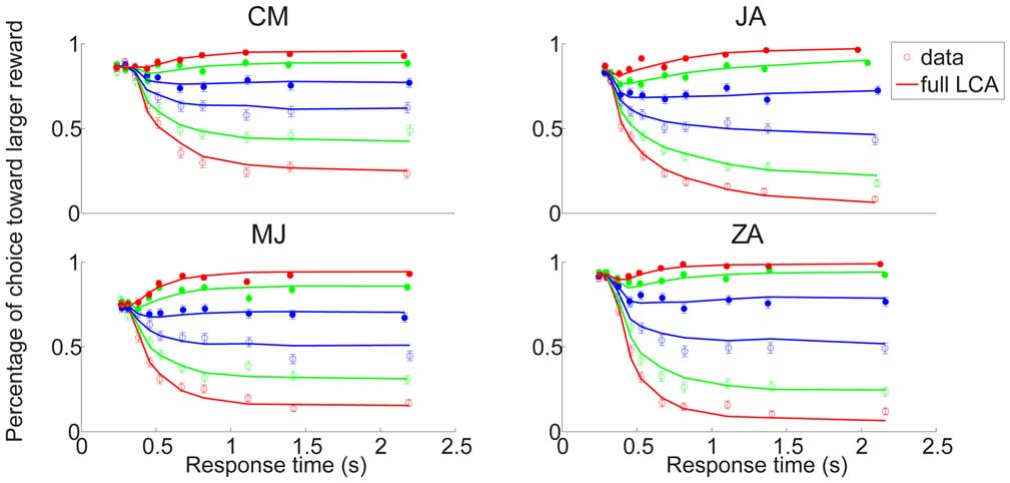
Fitting results under the initial condition hypothesis 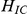, based on the full nonlinear leaky competing accumulator model. Figure format is as in Figure 10. See Table 3 for fitted parameters.

The expected response probability patterns for each of the participants are plotted in Figure 11 together with the data. Evidently, with the chosen values of the 2 additional parameters, the full two dimensional model fits the experimental data in a way that is very similar to the fit provided by the reduced model. Overall, it appears that 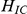, in which reward offsets the initial condition of the decision process, provides a very good account of the observed data.

## Discussion

In this work, we attempted to answer the question: How do humans integrate reward and stimulus information dynamically in perceptual decision-making? We used a perceptual decision-making task with unbalanced rewards in which stimulus duration varied from 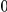 to 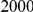 ms. We found that, for four of the five participants in the experiment, reward bias, measured in terms of the position of the normalized decision criterion 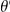, starts high initially and declines as stimulus sensitivity builds up, then levels off as stimulus sensitivity reaches asymptotic levels.

We find that the detailed pattern of results can be captured by the inhibition-dominant leaky competing accumulator model (LCA), under the assumption that reward offsets the initial state of the accumulators before stimulus information begins to accumulate. In the inhibition-dominant regime, the accumulator that has an initial lead in activation tends to suppress the other accumulator. The advantage thereby granted by the reward difference to the accumulator associated with the higher reward actually builds up over time, although it lessens in a relative sense as time goes on since variability builds up even more quickly initially. The model explains how an imbalance in the initial activation of the two accumulators can produce a monotonically decreasing shift in the position of the normalized decision criterion, as seen in the data, in terms of the relative rates of growth of the reward offset signal and the total accumulated noise. It is worth noting that our analysis revealed that the three different accounts of the way in which reward might affect the information integration process each has its own qualitatively distinct empirical signature. Thus, we were able to rule out two of the three hypotheses by relying on the qualitative form of the data. Focusing on the remaining hypothesis, we found it to provide not only a match to the qualitative pattern of the data but also to allow a close fit of the exact quantitative pattern in the data as well.

Our use of the inhibition-dominant LCA is not strictly required by the present data – these data could be fit by a leak-dominant variant of the model equally well (See Appendices 2 and 3). Our choice to pursue the inhibition-dominant regime is not arbitrary, however. It is based on findings of other recent studies using similar paradigms, in which humans or primates must be prepared to respond quickly to an imperative go cue or response signal, as they must in our experiment. The inhibition-dominant LCA simultaneously explains (a) why accuracy levels off at non-ceiling levels as stimulus processing time increases, and (b) why information coming early in a trial exerts more influence on decision outcomes than information coming later [22], [23].

### Alternative Models

While the model offers an excellent fit to the data, this does not necessarily preclude the possibility that other approaches might also be able to explain the present data. Future research will be needed to examine the full range of possible alternative models. Here we briefly consider whether our results can be explained within the classic drift diffusion model.

The first point to note is that the drift-diffusion model, in its simplest form (no between trial drift variance and no bound in the integration of information) predicts that accuracy will continue to grow without limit, something that is not observed in this or other experiments. The leveling off of accuracy as a function of processing time can be explained by assuming there is trial-to-trial variability in the driving input to the evidence accumulation process [2]. While such an approach can provide a good fit to our stimulus sensitivity data, it is not consistent with the pattern of reward bias effects we observe under any of the hypotheses we have considered. Under either the initial condition hypothesis 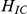 or the fixed offset hypothesis 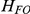 the effect of the reward bias will eventually become negligible, because the variance of the evidence accumulation process increases without limit. Under the ongoing input hypothesis 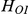, in which the reward input starts with reward cue onset and continues until the response choice is initiated, it is possible to capture a large initial bias that reduces as accuracy grows and then levels off. However, according to such a model, the fit is constrained by the fact that the normalized reward bias and the stimulus sensitivity have the same dynamics except that reward starts 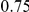 second earlier. For example, in order to prevent the stimulus sensitivity from saturating too early, the main source of noise should be within-trial variability. However, this results in a dip in the normalized reward bias curve as in 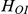 in the 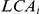 framework. Due to these constraints, the fit to the data is poorer than the fit to the 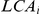 for all four participants (log-likelihood values are 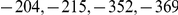 for CM, MJ, JA, and ZA, respectively; fits were obtained using an unbounded drift diffusion model with free parameters for sensitivity 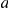, overall reward strength 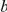, between-trial drift variability 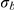, starting point variability 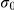, and dead-time parameter 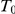, amounting to the same number of free parameters as the 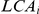).

We considered the further possibility that the imposition of a bound on the integration process might allow the DDM to account for our data, since it has been argued that participants may reach a integration bound before the go cue occurs [22]. However, inclusion of a bound tends to compromise the fit to the sensitivity data: the presence of the bound tends to cause 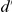 to asymptote earlier for easy trials and later for hard trials, contra the pattern in the data. It remains possible that some version of the drift diffusion model could account for the data. We leave it to others to explore this possibility further.

Our findings on the effects of reward bias are largely consistent with, but also extend, other recent studies of the role of reward in the dynamics of decision-making. Previous human behavioral studies [12], [13] also rejected the idea that reward bias acts as a continuing input to the state of the accumulators during accumulation of stimulus information, and supported a two-stage model, similar to ours in some ways, in which processing of reward cues preceded, and set the initial state, of the evidence variable prior to the start of stimulus processing (for further consideration of this model, see *future directions* below). Neurophysiological data from [15] likewise supports the view that reward cues affect the starting point of an information accumulation process in a paradigm somewhat similar to ours, albeit one with a fixed stimulus duration. Neither study explored as wide a range of processing times as the present study, and in consequence, neither study showed clearly that reward bias effects decrease but level off at nonzero levels as processing continues.

Consistent with findings in [14], for the four participants who showed reward bias effects, the amount of bias is close to optimal when stimulus duration is long, deviating slightly and with relatively little cost in the under-biased direction. In contrast, when they have zero sensitivity to the stimulus at very short delays, all participants deviate from the optimal strategy of always choosing the alternative associated with the higher reward. We should also note that one of the five participants in the study failed to exhibit a systematic reward bias. We have occasionally seen this pattern in other participants tested on variants of the task used here, and the finding is reminiscent of the finding in the study of Diederich and Busemeyer [13], in which there was a group of participants who showed little or no sensitivity to their reward manipulation. We have sometimes found that participants could be induced to show reward bias effects through persistent instructions reminding them of the benefits of exploiting reward information when stimulus information is uncertain, but we did not employ this approach in the present investigation.

### Explaining the Initial Underbias in Choice Responses

**Figure 12:**
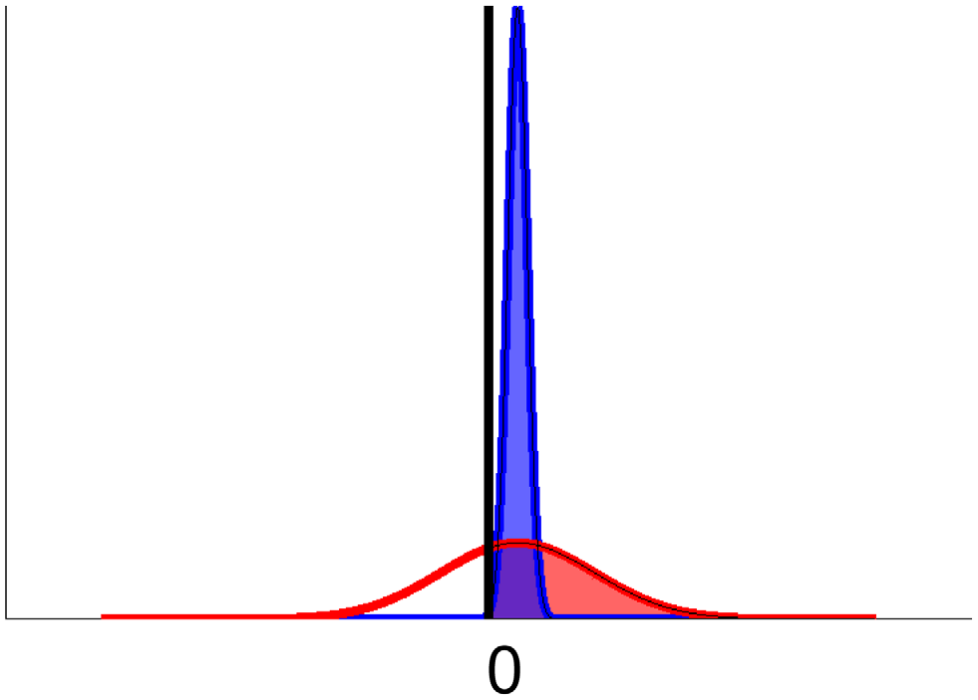
The magnitude of the effect of reward bias is affected by the initial variability. The same reward offset (center position of the distributions relative to 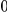) results in choosing the higher reward alternative almost 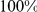 of the time when the variability in the activation difference variable is very low (blue); while this occurs much less often (about 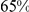 of the time in the case illustrated) when the variability is high (red).

Can our model help explain why participants do not always choose the higher reward alternative at short processing times, where performance shows no stimulus sensitivity? In the model, one factor that limits the size of the initial reward bias is initial variability in the activations of the accumulators (see Figure 12). This initial variability may reflect a carry over or compensation for previous trials [29], and it can also reflect noise accumulated within a trial before stimulus onset. The variability could also arise from trial-to-trial fluctuation in the magnitude of the reward offset signal. For the same amount of mean offset in the activation difference variable due to reward, the resulting effect on response probability is strongly affected by this initial variability. Indeed, if the initial variability were 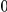, even a very slight initial reward bias would always lead to a choice of the higher reward alternative in our model.

The initial variability, as well as other parameters associated with each individual participant's performance, might be viewed as inherent in the decision process – factors the participant has little or no ability to control. However, even if the amount of initial variability and these other parameters were fixed, a decision maker could still come closer to achieving an optimal bias on short trials by offsetting the activation difference variable by an even larger amount. Assuming participants have strategic control over the magnitude of this initial bias, the question then arises, why do they not simply make the initial bias even stronger?

**Figure 13:**
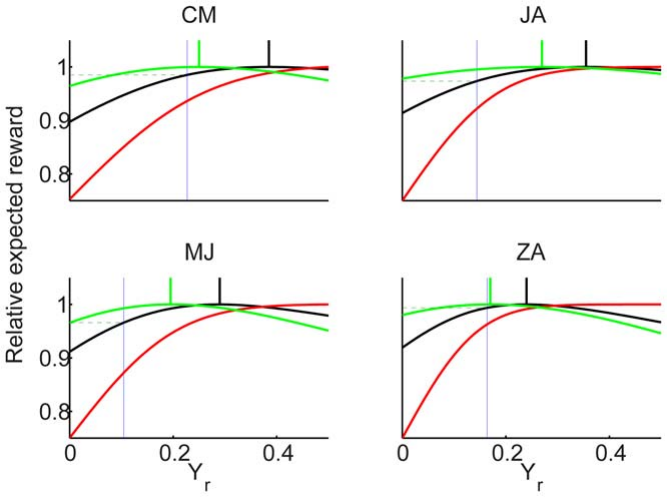
Normalized expected rewards as a function of the reward offset under the initial condition hypothesis. Expected reward obtained in the shortest delay condition (red), the longest delay condition (green) and all delay conditions (black) are plotted together. In each condition, the amounts of reward are normalized by the maximum rewards the participant can achieve in that condition or set of conditions. In each panel, the vertical blue line denotes the observed reward offset, the vertical green bar on top of the green curve indicates the amount of reward offset that would optimize performance for the longest duration and the vertical black bar on top of the black curve denotes the amount of reward offset that maximizes the overall expected rewards across all durations. Note that optimal reward offsets are based on the fits of the model rather than on the data. See text for details.

One response to this question is to note that if participants offset the starting point of the accumulation process by too much, this may produce an over-bias on trials where the stimulus duration turns out to be very long. To investigate this, we can examine, for each participant, the expected rewards for different delays, and for the average over delays, as the magnitude of the initial reward offset increases (Figure 13), holding all other parameters of the decision process constant. The amount of offset that maximizes reward in the longest delay condition (the vertical green bar on the top of the green curve) is plotted together with the amount of offset each participant used (vertical blue line), according to the fitted value of the reward offset parameter in the one dimensional reduction of the model. Also shown (vertical black bar on top of the black curve) is the amount of bias that will optimize reward overall. This plot demonstrates that the actual bias is close to optimal for longer delays, but that all participants will gain more rewards overall by starting each trial with a larger reward offset.

Why participants do not fully optimize the magnitude of their reward bias is a question that cannot be fully resolved by the present study. However, it may be worth considering a few possibilities. One possible reason could be that participants' subjective utility is a decreasing function of the objective rewards [30], [31]. The desirability of winning 2 points may be less than twice that of winning 1 point, or alternatively, observers may place some intrinsic value 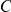 on being correct, independent of the reward, such that the subjective reward ratio becomes 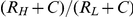; this quantity is always less than 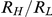 as long as 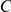 is positive. See also [32] and references therein. Another possibility is that setting a large initial offset in the accumulators requires effort, and participants are trading off a small amount of their possible payoff for a reduction of this effort. In this connection it is worth noting that all four of the participants who showed reward effects are achieving within 5% of their maximum possible reward in the longest delay condition (as indicated by the horizontal dashed lines on each panel of Figure 13). Thus, even if the extra effort required were only moderate, the benefits might not be worth it. This is due to the shallow curvature of the reward harvesting curves, especially for the long delay conditions. Similar shallow reward curves are reported in the free response protocol with the drift diffusion model [33].

Beyond these possibilities, there are other kinds of reasons why participants might not adopt a larger reward offset: One possibility that is of interest from the point of view of models like the LCA is that too large a reward offset would distort the dynamics of the information integration process. As things stand, according to the analysis offered by our model, the dynamics of the decision-making process are effectively linear, in the sense that the linear approximation offered by the one dimensional simplified model provides a close fit to the data. A larger offset might push the dynamics of the process outside of the region where this approximation holds, and into a regime where the nonlinearity in the process would lead to deviations from optimality. A further possibility is that there are nonlinearities at the upper end of the activation range not fully captured in either version of our model, but present in more biologically realistic models [7], and that these would come into play with strong initial reward biases. Also, if we view the accumulators in the model as neural populations that actually trigger overt responses when their activation reaches a critical level (as the squeeze of a trigger causes a gun to fire), then too much activation of an accumulator might actually trigger overt responding prematurely. In that case, participants would have to limit the magnitude of the initial activation of the more highly rewarded alternative. As previously noted, selection among these possibilities is beyond the scope of the present paper.

### Open Questions and Future Directions

Here we consider several further issues that remain open and discuss some possible directions for further research on these matters.

We have provided an account for the role of reward bias in a particular paradigm, and the account provides quite a good fit to the data from all four participants. There may be room for further improvement, however, in the adequacy of the fit in two of the four cases. One obvious question is to explore how other models would fare in fitting these data, and also to investigate whether an even better fit might be achieved within the 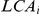 framework. In examining the pattern of deviations from the fit offered by the current version of the inhibition-dominant leaky competing accumulator (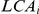) model, we see little clear pattern in the case of participant MJ, and so are uncertain whether a closer fit will be possible with any parsimonious model. In the case of participant ZA, however, the deviations appear to reflect a slight under-representation, on the part of the model, of the degree of reward bias in the hardest stimulus condition (both blue curves fall above most of the corresponding data points). Otherwise, the fit appears to capture other features of the data quite accurately. Whether a slight adjustment of the current model, or some alternative model, is able to capture this small but apparently systematic deviation is an issue that should be explored in further research. More generally, we welcome comparison of the account offered by the 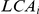 to other possible approaches to capturing the overall pattern in the data.

Several broader questions, going beyond the details of our specific experiment, also deserve to be examined in future studies. One concerns how well the 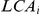 might explain the pattern of data presented in the two studies mentioned earlier on reward bias effects in a task that is similar to ours in many respects but relies on a deadline procedure [12], [13]. The models considered in those papers did not include leakage or inhibition. Two models that share with our model the assumption that reward affects the initial state of the accumulators were considered in these papers, although the modeling framework used could not distinguish between an offset in the starting place of the accumulators *per se vs.* an offset in decision criteria. (One of the models considered in both [12], [13]–the ‘two-stage’ model– is most naturally viewed as a model in which the first (reward-processing) stage drives activation of the accumulators, but it is still possible to think of this stage as one that introduces a complementary adjustment in the position of decision boundaries). Although some of the models considered provided better fits to the data than others, there was still room for improvement even for the best models considered. In light of this, it will be interesting to see how well the 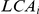 may be able to account for the data from these studies.

Reward effects might also be explored in a standard reaction time experiment, in which no explicit time constraint on processing is provided. In such experiments, participants are usually thought to respond when the activation of one of the detectors reaches a criterial activation level. In the absence of trial-to-trial variability in the input to the accumulators, the optimal policy in the classical diffusion model is to offset the starting point of the accumulators (or equivalently, to offset the positions of the decision boundaries) by a fixed amount. However, if there is trial to trial variability in stimulus difficulty (either due to drift variance or to a mixture of difficulty levels), a superior policy may be to allow the amount of reward bias to gradually increase [34], or, alternatively, to allow it to produce a gradual decrease in the position of the decision boundaries. This will have the useful consequence of leading to less reward bias for the easy conditions (which will tend to reach a boundary early) compared to the harder conditions (which will tend to reach the boundary later, when the effect of the bias is greater). It will be interesting to see whether participants are able to achieve near-optimal reward bias effects under such conditions, and if so to understand how such effects are implemented mechanistically.

Additionally, further research is needed to investigate the neural basis of reward effects on the dynamics of decision-making. While the Rorie et. al. study [15] provides important evidence on this issue, in a paradigm that has many similarities with the one we have used in these studies, it would be desirable to develop non-invasive methods for use in human studies as well, preferably using imaging modalities such as EEG and MEG with high temporal resolution. Investigations of this type are currently in progress in our laboratory.

Another important direction for future investigations is to understand better the individual differences we see between participants, and to discover ways in which participant's performance can be optimized. In the earlier part of this discussion, we focused on optimization of the way in which the reward bias influences the decision-making process, considering other parameters as fixed, but it may be that other parameters of the process are also subject to strategic control, and hence possible optimization. Participants may have some control over the variability in the initial state of the accumulators. For example, they may be trying to anticipate which alternative will be presented on a given trial, even though this is completely randomly determined. Alternatively, participants may have some control over the shared input to the two accumulators (the 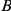 parameter in the full two dimensional model), and/or the balance between leak and inhibition. These parameters might be affected by top-down activation signals or by neuromodulatory processes partially or completely under strategic control, or at least subject to individual differences. Exploration of these possibilities will be an important target of future investigations.

### Conclusion

Our investigation has considered how reward information affects decision dynamics under conditions of time pressure and uncertainty, and we have found that all four of the participants who exhibited sensitivity to reward information showed a pattern of reward bias in which responses after very short processing times exhibited a strong reward bias, which tapered off to a steady level as stimulus sensitivity also approached an asymptotic level. A good account of our data was provided by a variant of the leaky competing accumulator model, in which reward offsets the starting place of a competitive, inhibition-dominant, activation process. Exploring this further within the model, the initial offset values fitted to the data of all four reward-sensitive participants allowed each of them to harvest more than 95% of possible rewards, fixing all other parameters of the model. Additional research is needed to determine why participants were not able to come even closer to optimality. Further research is also needed to consider how well our data might be explained by alternative models; to further understand the role of reward in other paradigms in which responses must be made quickly based on uncertain information; to understand the neural basis of reward effects on decision-making; to understand individual differences; and to explore the extent to which other aspects of participants' performance in tasks requiring the integration of reward and stimulus information can be optimized.

## Methods

The research on human participants reported herein was approved by the Stanford University Institutional Review Board (nonmedical subcommitte) under protocol #7029. Written informed consent was obtained from all participants.

The stimulus was displayed on a Dell LCD monitor at 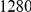
×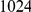 resolution using the Psychophysics Toolbox v2.54 extensions of Matlab r2007a. All stimuli were light gray rectangles on a darker gray background. On each trial, the rectangle was longer to the left or right of the screen center by 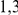 or 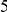 pixels over a basis of 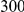 pixels, resulting in a shift of the center by 0.5, 1.5, or 2.5 pixels. Participants, seated approximately 2.5 feet from the monitor, were asked to judge which side of the rectangle was longer and to indicate their decision by pressing one of two specified keys assigned to the left and right index fingers.

On each trial, participants saw a fixation cross for 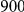 ms. An arrow then replaced the fixation cross for 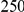 ms, pointing either left or right to indicate which of the two responses, if correct, would lead to a 2-point reward. The other alternative was always associated with a reward of one point. The arrow was then replaced by the fixation cross, and a stimulus was displayed 500 ms later. After the stimulus appeared, participants were instructed to hold their response until they heard the “go” cue. The cue tone was played 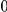 to 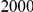 ms after the appearance of the stimulus. There were ten possible cue onset times within this range. Participants were to respond within 250 ms of the onset of the go cue.

The stimulus disappeared after the response. Visual and auditory feedback was given 750 ms after the go cue indicating whether the response occurred within 250 ms, and (if so) whether it was correct. If participants responded within 250 msec and chose correctly, they heard a cash register sound (‘ka-ching!’) once or twice, and earned either 1 or 2 points. A correct response in the direction indicated by the arrow would earn two points, while a correct response in the opposite direction would earn only one point. Incorrect responses earned no points and were followed by an error noise. Responses that occurred too early or too late also received no points, and were followed by a different noise.

**Figure 14:**
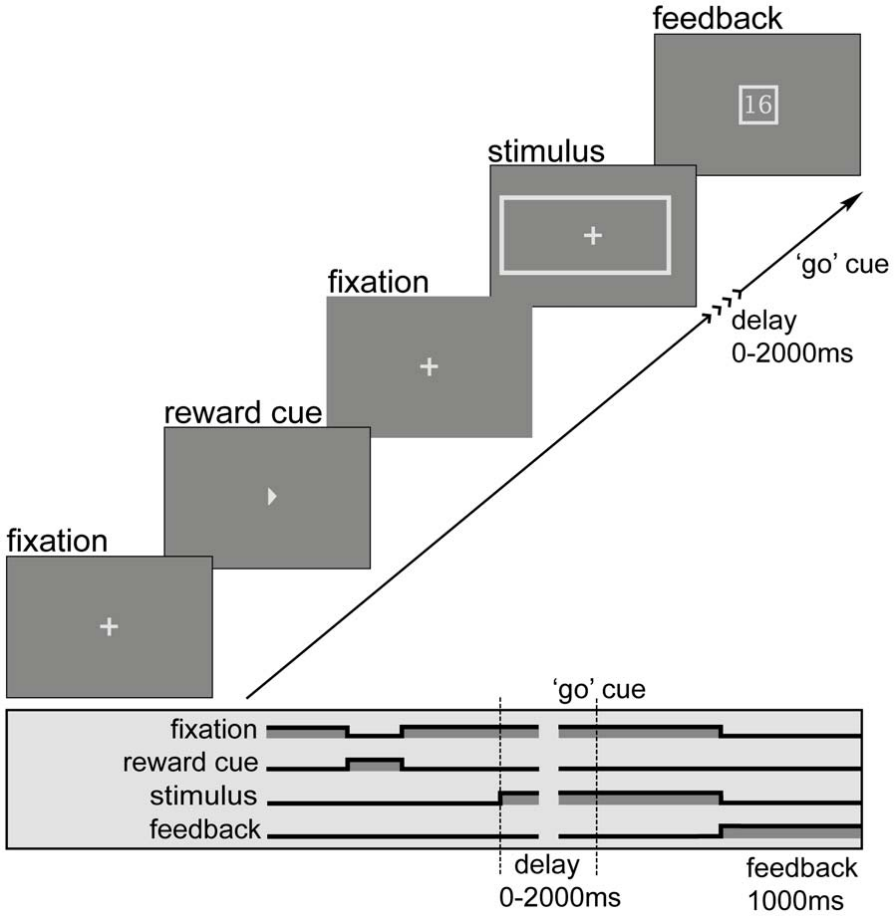
Procedure of the perceptual decision-making task with unequal payoffs. The reward cue (a left or right pointing triangle) indicates which choice, if correct, receives higher reward. Timing of the stages of the experiment is depicted on the bottom. The “Go” cue comes on with a delay of 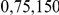, 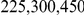, 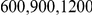 or 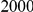 milliseconds. See text for details.

The total time allotted for feedback of any type was 1000 ms. Participants were paid a base amount of USD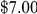 per session and an additional amount equal to 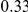 cent per point earned. See Figure 14.

Five participants who reported normal or correct-to-normal vision and hearing were tested in one-hour sessions over several weeks. In each session, all combinations of discriminability level (1, 3, 5 pixels longer to the left or right), reward (left- or rightward arrow) and delay condition (“go” cue occurring 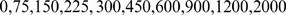 milliseconds after stimulus onset) were presented in a pseudorandom manner. In each session, participants completed 7 blocks of 126 trials. A self-timed break occurred between blocks. For all participants, the first two sessions in which they familiarized themselves with the task were ignored. The total number of trials included in the reported analysis were 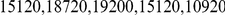 for participants CM, JA, MJ, ZA, and SL respectively.

## Supporting Information

Supporting Information S1 Here, we present the Iso-Criterion analysis of the data. For each delay condition, we plot the stimulus sensitivity and the decision criterion variable representing the degree of reward bias individually for each of the three difficulty levels. The results are generally consistent with the hypothesis that the participants are adopting a common criterion for the three difficulty levels within each delay condition.
(PDF)

Supporting Information S2 In the linear version of the leaky competing accumulator model, exactly the same pattern of choice behavior can be predicted in either leak- or inhibition-dominance with proper parameter values. Here, we demonstrate this result and show the relationship between the two parameter sets in the two regimes.
(PDF)

Supporting Information S3 Here we consider how reward might influence choice behavior in the leak-dominant regime of the leaky competing accumulator model, examining the same three hypotheses considered in the main text for the inhibition-dominant regime. Although the data from the reported experiment are treated as arising within the inhibition-dominant regime, we include this analysis to complete the analysis of the full theoretical framework.
(PDF)
