# Supplementary material for: Dynamic Integration of Reward and Stimulus Information in Perceptual Decision-Making
Source: PLoS One. 2011 Mar 3;6(3):e16749. doi: 10.1371/journal.pone.0016749 (PMC3048391; doi:10.1371/journal.pone.0016749)
Supplement: Supporting Information S1 — Here, we present the Iso-Criterion analysis of the data. For each delay condition, we plot the stimulus sensitivity and the decision criterion variable representing the degree of reward bias individually for each of the three difficulty levels. The results are generally consistent with the hypothesis that the participants are adopting a common criterion for the three difficulty levels within each delay condition. (PDF) [file pone.0016749.s001.pdf]

Supporting Information S1: Criterion Analysis: Iso-Criterion Lines

Researchers working in the framework of signal detection theory have noted that it is often useful to graph data on a z-transformed plot, where each condition of an experiment is represented by a single point, corresponding to the z-transform of the 'hit rate' in a given condition plotted on the y axis against the z-transform of the 'false alarm' rate plotted on the x axis. To apply that approach to our data, we treat the probability of choosing the high reward response when the stimulus is in the direction of the high reward as corresponding to the hit rate and the probability of choosing the high reward response when the stimulus is in the opposite direction as corresponding to the false alarm rate. For each participant, we plot a separate point for each difficulty level, S = 1, 3, or 5. Under the assumption that evidence variable is distributed normally with the same standard deviation for each of the possible stimuli (+-1, +-3, +-5), the distance of the point from the positive diagonal represents the value of d'_i for that difficulty level, and the distance of the point from the negative diagonal represents the value of θ'. Under the further assumption that a fixed criterion is used within each delay condition, the points for the three difficulty levels at a given delay should fall on a single *iso-criterion line* with slope -1, shifted by the distance θ' from the negative diagonal. In Figure S1.1, we plot the data in this way, using a separate panel for each participant. Also shown are the corresponding iso-criterion lines for each delay condition. Visual inspection reveals, as expected, that the series of iso-criterion lines start relatively far from the negative diagonal for the shortest delay conditions, and shift with delay toward the negative diagonal, but do not reach it, consistent with the fact that a bias toward the high reward remains even at the longest delays. Generally speaking the data points fall near the corresponding iso-criterion line. The fact that sensitivity increases with delay is reflected by the movement of the points away from the positive diagonal (representing increasing d').

To assess whether the three d' values and one θ' value provide an empirically adequate characterization of the data, we conducted *Chi square* tests as follows. For each participant, in each of the 10 delay conditions, we compared the observed probability of choosing the high reward response in each of the six stimulus conditions to the values that would be expected given the three d' and one θ' value. Each such test is a *Chi square* test with 2 degrees of freedom (six independent data points fitted using four free parameters). We found only one instance in which the *Chi square* value approached significance (smallest p value = 0.057). Out of 50 independent tests, one would expect two or three should have p values less than .05 by chance. Thus, it appears that the three d'_i values and single θ' value provide a reasonably good empirical description of the data.

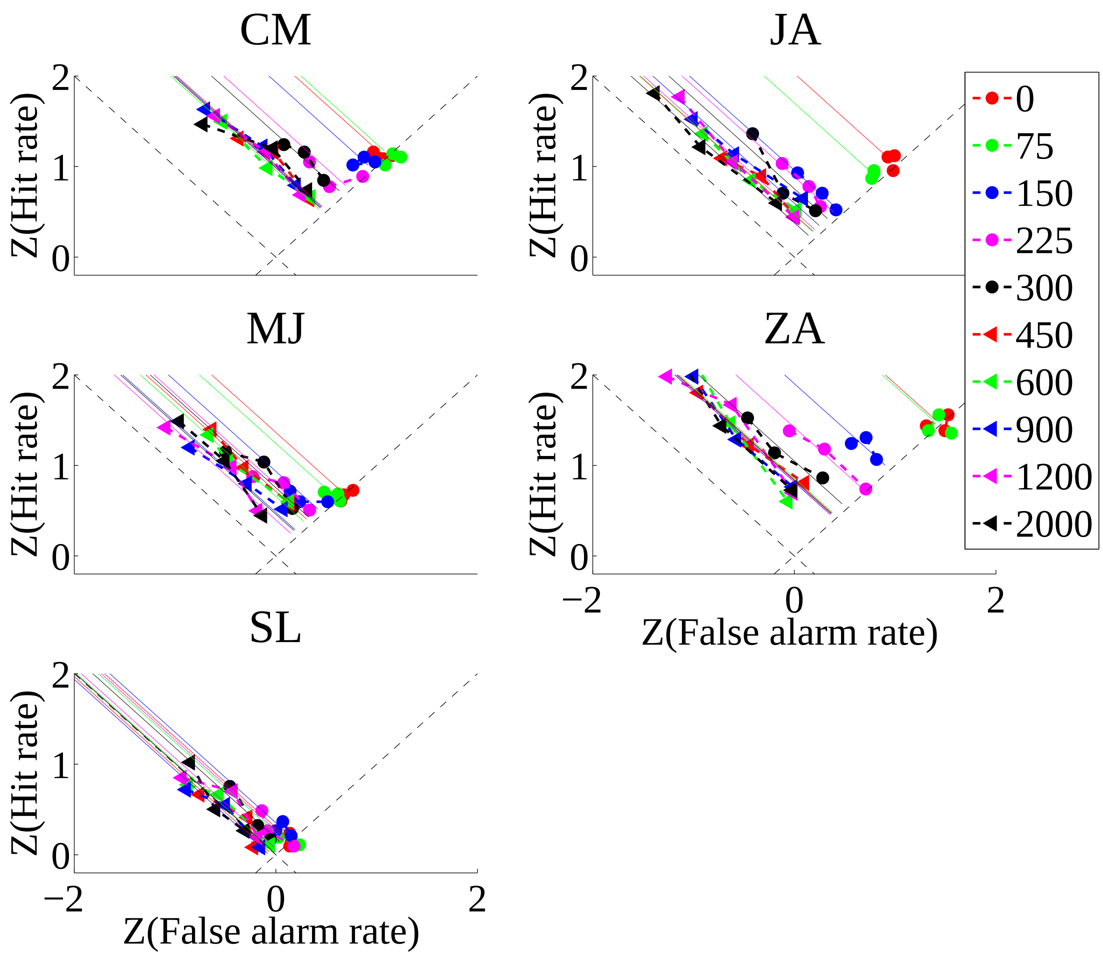

Figure S1.1. Iso-criterion analysis of the response probability results. For each delay condition, the z-transform of the hit rate (probability of choosing the higher reward alternative in the congruent condition) is plotted against the z-transform of the false alarm rate (probability of choosing the higher reward alternative in the incongruent condition) for each of the three difficulty levels. See text for details. Delay conditions are color- and symbol- coded. Mean bias level for an individual delay condition is plotted as a thin line in the corresponding color.
