# Supplementary material for: Dynamic Integration of Reward and Stimulus Information in Perceptual Decision-Making
Source: PLoS One. 2011 Mar 3;6(3):e16749. doi: 10.1371/journal.pone.0016749 (PMC3048391; doi:10.1371/journal.pone.0016749)
Supplement: Supporting Information S2 — In the linear version of the leaky competing accumulator model, exactly the same pattern of choice behavior can be predicted in either leak- or inhibition-dominance with proper parameter values. Here, we demonstrate this result and show the relationship between the two parameter sets in the two regimes. (PDF) [file pone.0016749.s002.pdf]

Supporting Information S2: Same Response Probabilities in Leak- and Inhibition-Dominance

In this appendix, we show that exactly the same choice behavior can be predicted in either leak- or inhibition-dominance with proper parameter values. Given a parameter set in one situation(e.g. the parameters in leak-dominance), the corresponding parameters in the other situation (inhibition-dominance) can be obtained by simple linear scaling. We focus on basic decision dynamics without reward bias in this section and proceed to the discussion of reward bias in the following section.

As we emphasized in the main text, response probability in both leak- and inhibition-dominance is determined by the ratio between the mean and the standard deviation of the activation difference variable. See Equation (8) in the main text. For the leak-dominant case, we denote the values of the timescale λ, personal sensitivity a and the initial variability σ_0 with L_λ, L_a and $L_{\sigma 0}$ respectively. For the inhibition-dominant case, we denote the corresponding parameters with I_λ, I_a and $I_{\sigma 0}$. By assigning the parameter values such that

$$I_\lambda = -L_\lambda; I_a = \frac{L_a}{\sqrt{1 - 2L_\lambda L_{\sigma 0}^2/\varepsilon^2}}; I_{\sigma 0} = \frac{L_{\sigma 0}}{\sqrt{1 - 2L_\lambda L_{\sigma 0}^2/\varepsilon^2}},$$

we will find that the accuracy dynamics Equation (8) turns out to be the same with the leak and the inhibition parameter sets.

This means that exactly the same response probability data can result from leak- or inhibition-dominance with the same absolute value of λ and linearly scaled personal sensitivity a and initial variability σ_0. The common scalar of a and σ_0, $\kappa = \sqrt{1 - 2L_\lambda L_{\sigma 0}^2/\varepsilon^2}$ has its own meaning and will appear again. Without initial variability, the variance of the activation difference variable in leak-dominance grows from 0 and saturates at $\sqrt{\varepsilon^2/2L_\lambda}$. With initial variability, it starts from $L_{\sigma 0}$ and saturates at the same value. The growth range of the variability is hence shrunk, and the scalar κ is the shrinking rate. In the special case of zero initial variability, the scalar becomes 1. Otherwise, $\kappa < 1$, implying that to produce the same choice behavior, the initial variability and the personal sensitivity should be relatively smaller in leak-dominance.
