# Supplementary material for: Dynamic Integration of Reward and Stimulus Information in Perceptual Decision-Making
Source: PLoS One. 2011 Mar 3;6(3):e16749. doi: 10.1371/journal.pone.0016749 (PMC3048391; doi:10.1371/journal.pone.0016749)
Supplement: Supporting Information S3 — Here we consider how reward might influence choice behavior in the leak-dominant regime of the leaky competing accumulator model, examining the same three hypotheses considered in the main text for the inhibition-dominant regime. Although the data from the reported experiment are treated as arising within the inhibition-dominant regime, we include this analysis to complete the analysis of the full theoretical framework. (PDF) [file pone.0016749.s003.pdf]

Supporting Information S3: Predictions of the Hypotheses in Leak-Dominance

In this appendix, we explore the predictions of the three hypotheses for the role of reward under the leak-dominant regime of the leaky competing accumulator (LCA) model. Although evidence cited in the main text tends to weigh against leak-dominance as an account for our data, we include discussion of the predictions in leak-dominance to provide a more complete analysis of possible effects of reward within the LCA framework.

Solutions of the activation difference variable and effects of reward in the three hypotheses are described with the same equations discussed in the main text. See Equations (7-14). However, λ is positive, in which case the values of the equations differ qualitatively from those in inhibition-dominance and the predictions are thus different. As in Figure 9, the dynamics of the activation difference variable and the reward effect are depicted in Figure S3.1. The top row demonstrates how the distributions of the activation difference variable evolve with time. The middle row depicts the reward effect on the mean of the distributions (green) and the width of the distributions (magenta). The bottom row shows the ratio between the two (i.e. the reward effect on the *normalized* decision variable), which represents the effect of the reward bias on response probability.

The ongoing input hypothesis H_{OI} in leak-dominance behaves the same way as it does in inhibition-dominance. As processing time lengthens, the reward bias declines first and then starts to climb. See bottom left panel of Figure S3.1. In fact, we obtain exactly the same response probability results if we assign the values of the parameters in leak- and inhibition-dominance accordingly. Taking the values of λ, I_r, σ_0 in inhibition-dominance and denoting them as $I_\lambda, I_I r, I_{\sigma 0}$, we can calculate their values in leak-dominance by a linear scaling:

$$L_\lambda = -I_\lambda; L_{Ir} = \frac{I_{Ir}}{\sqrt{1-2I_\lambda I_{\sigma 0}^2}}; L_{\sigma 0} = \frac{I_{\sigma 0}}{\sqrt{1-2I_\lambda I_{\sigma 0}^2}}.$$

Plugging these new parameter values into Equation (12), we will find the expression becomes the same as with the parameter set in inhibition-dominance. Note that the scaling factor $\kappa = \sqrt{1-2L_\lambda L_{\sigma 0}^2}$ is the same as that defined in the previous section, with the strength of the incoming noise ε set to 1, consistent with the presentation of the reward bias results under inhibition dominance, as discussed in the main text.

Under the initial condition hypothesis H_{IC} in leak-dominance, the reward effect on the activation difference variable (the numerator in Equation (13)) decays to 0 as stimulus duration lengthens. See solid green line in the middle panel in Figure S3.1. Since the accumulated noise (the denominator in Equation (13)) increases and saturates, the resulting reward bias on choice hence also decays away (bottom middle panel). Intuitively, this reflects the fact that influences on the activation state of the system generally decay away in the leak-dominant regime, including influences affecting the initial state of the system. The reward information, which only affects the starting point, hence disappears as processing time lengthens. Interestingly, the resulting effect of reward bias on choices under H_{IC} in leak-dominance shares the same properties with that of fixed offset hypothesis H_{FO} in inhibition-dominance. Mathematically, the same choice behavior will result if we take the parameters from H_{FO} in inhibition-dominance and assign parameter values under H_{IC} in leak-dominance through a scaling by κ. However, the underlying mechanisms are different. Under H_{IC} in leak-dominance, the reward offset itself decays to 0 with time; while under H_{FO} in inhibition-dominance, the reward bias disappears because the reward offset is scaled out by the exploding accumulated noise.

Under the fixed offset hypothesis H_{FO} in the leak-dominant regime, the numerator of Equation (14) remains constant, while the denominator (the accumulated noise) grows and saturates. See the solid green and magenta curves in the middle right panel in Figure S3.1. The resulting ratio hence starts from a non-extremal value and declines to a nonzero value. See the bottom right panel. This pattern

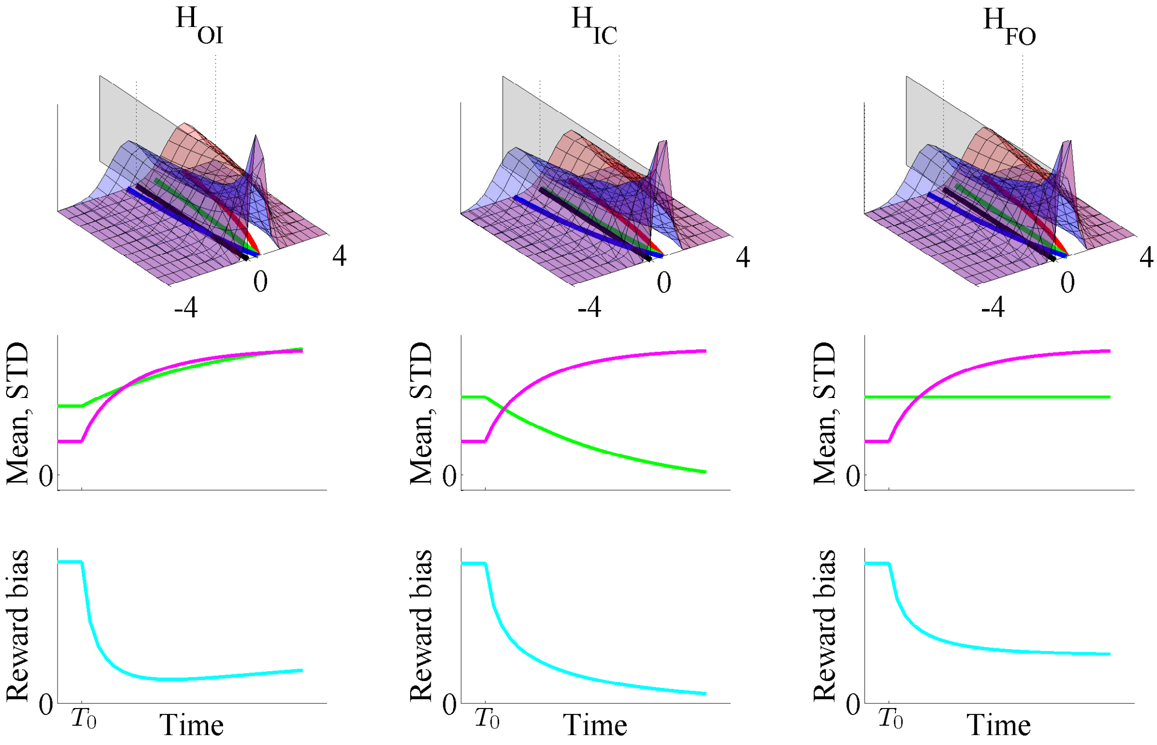

Figure S3.1. Reward effects in the three hypotheses in the leak-dominant regime. The figure is formatted as in Figure (9) in the main text. Note that the patterns of the reward bias of the three hypotheses in leak-dominance differ from those in inhibition-dominance: Under H_{IC}, like under H_{FO} in inhibition-dominance, reward bias on choice disappears with time; under H_{FO}, like under H_{IC} in inhibition-dominance, the reward bias sustains with time. The predicted reward bias of H_{OI} in leak-dominance is similar to that in inhibition-dominance.

is the same as the prediction of H_{IC} in inhibition-dominance. Quantitatively, we can again scale the parameters under H_{IC} in inhibition-dominance to obtain the same response probability under H_{FO} in leak-dominance.

The above theoretical explorations revealed that the consequences of particular ways of implementing a reward bias effect differ between the leak-dominant and inhibition-dominant regimes. In related work, we are exploring whether task variables, such as the requirement to respond immediately after presentation of the go cue, affect decision dynamics. If so, this would have implications for the implementation of reward bias effects when the task characteristics are changed.
